# Phenolation of cyclodextrin polymers controls their lead and organic micropollutant adsorption†
†Electronic supplementary information (ESI) available: Experimental procedures, Pb^2+^ binding isotherms and affinity plots, ^1^H, ^13^C, and ^19^F NMR spectra, FT-IR Spectra. See DOI: 10.1039/c8sc03267j


**DOI:** 10.1039/c8sc03267j

**Published:** 2018-09-24

**Authors:** Max J. Klemes, Yuhan Ling, Marta Chiapasco, Alaaeddin Alsbaiee, Damian E. Helbling, William R. Dichtel

**Affiliations:** a Department of Chemistry , Northwestern University , Evanston , IL 60208 , USA . Email: wdichtel@northwestern.edu; b School of Civil and Environmental Engineering , Cornell University , Ithaca , NY 14853 , USA; c Department of Chemistry and Chemical Biology , Cornell University , Ithaca , NY 14853 , USA

## Abstract

Porous β-cyclodextrin polymers linked with tetrafluoroterephthalonitrile (TFN-CDPs) have shown promise for adsorbing organic micropollutants (MPs) more quickly and effectively than conventional adsorbents. Prior to their discovery, the nucleophilic aromatic substitution (S_N_Ar) reaction used to prepare TFN-CDP was nearly unknown for the aliphatic alcohol nucleophiles, and the low isolated yields of TFN-CDP motivated model studies of the reaction between TFN and *n*-butanol. These experiments reveal a previously undescribed substitution reaction of TFN in which a fluorine is substituted by a hydroxyl group. This process is responsible for the low yields of the polymerization and incorporates phenolate groups into the polymer network. Phenolation and polymerization (etherification) are competing processes, and the level of phenolate incorporation was controlled by varying the rate of base addition and initial monomer concentrations. TFN-CDPs with varying phenolate content were prepared and evaluated as adsorbents for both Pb^2+^ ions and 83 MPs. More heavily phenolated polymers showed increased capacity to bind Pb^2+^ ions. Phenolation was also correlated with increased binding affinity for almost all of the 83 MPs tested, including neutral, cationic, and anionic substances. These results leverage a newly discovered side reaction during S_N_Ar reactions of electron-poor aryl fluorides to improve both the yield and the uptake affinity for both lead and organic MPs of TFN-CDPs.

## Abstract 2

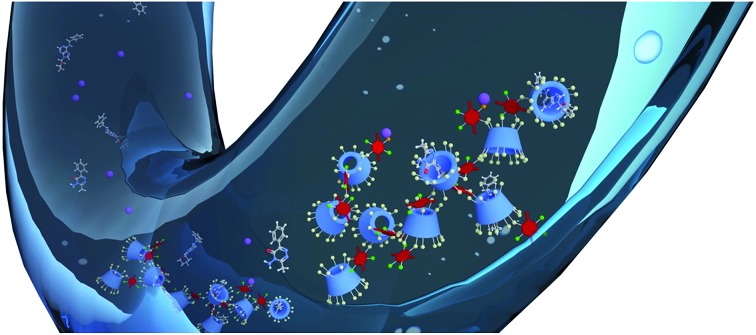
Lead and superior micropollutant sequestration by phenolated cyclodextrin polymer networks.

## Introduction

**Fig. 1:**
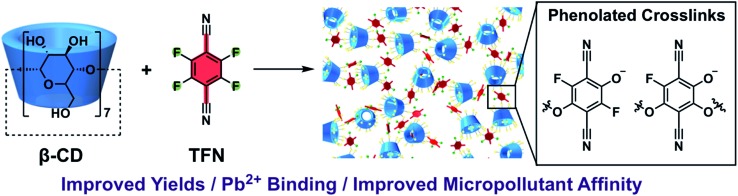
Synthesis of β-cyclodextrin (β-CD) polymers linked with tetrafluoroterephthalonitrile. This study identifies phenolated TFN-derived species incorporated into the polymer and characterizes their effect on Pb^2+^ and organic micropollutant binding.

The contamination of groundwater and surface water by heavy metals and organic micropollutants (MPs) is a world-wide problem.^1^–^4^ There is no safe level for lead in drinking water.^5^ Low concentrations of MPs (μg to ng L^–1^) can have deleterious effects in aquatic ecosystems,^6^–^8^ and long-term exposure to complex MP mixtures in drinking water may contribute to health and behavioral problems in humans.^9^ Currently, the economically scalable methods of water treatment for organic MPs are advanced oxidation^10^ and activated carbon (AC) adsorption.^11^ Advanced oxidation effectively reduces the concentration of organic MPs in water but can leave behind partially oxidized byproducts that retain toxic activity.^12^,^13^ AC adsorption effectively removes organic MPs but requires energy-intensive regeneration^14^ and can be fouled by natural organic matter.^15^ These problems might be addressed by using cyclodextrin (CD)-based adsorbents, which form well-defined host–guest complexes,^16^–^18^ are readily regenerated,^19^ and can exhibit rapid micropollutant removal.^20^ Cyclodextrin polymers and other porous, bio-derived materials have been used in water purification to remove pollutants including: phenolic molecules,^21^,^22^ dyes,^23^–^25^ naphthenic acids,^26^ perfluoroalkylated acids,^27^ and heavy metals.^28^,^29^ We previously reported porous polymer networks prepared *via* a nucleophilic aromatic substitution (S_N_Ar) polyaddition between β-CD and tetrafluoroterephthalonitrile (**TFN-CDP-1**).^30^**TFN-CDP-1** had a Brunauer–Emmett–Teller surface area of 260 m^2^ g^–1^ and exhibited more rapid uptake for many MPs compared to leading ACs and low-surface-area CDPs. The adsorbent was regenerated easily, was not fouled by humic acid in simulated surface water,^20^ and was further developed as a promising resin for solid-phase microextraction.^19^ However, the yield of the material obtained from the initial polymerization conditions was low (18%), and the TFN : β-CD ratio of the isolated polymer (*ca.* 6 : 1) did not match the monomer feed ratio (3 : 1).^30^ Furthermore, while polycondensations of TFN with catechols provide high molecular weight polymers that indicate efficient and selective reactions,^31^ there was only a single prior example of an S_N_Ar reaction between TFN and an aliphatic alcohol.^32^ Here we report a phenolation side-reaction of TFN that competes with its S_N_Ar reactions with aliphatic alcohols. Although this process contributed to the low yields of TFN-CDP in our previous report, we now balance the rates of phenolation and productive etherification reactions to improve polymerization yields and tune adsorbent performance (Fig. 1). Phenolated TFN-CDPs bind both organic micropollutants and Pb^2+^ ions more effectively than similar polymers with fewer phenolates. These findings demonstrate the promise and tunability of CD polymer networks to purify contaminated water. Identifying and controlling the loading of these phenolate functional groups in TFN-CDPs is therefore important for maximizing their performance and manufacturability. Furthermore, TFN is a common monomer in other classes of porous polymers^31^,^33^–^36^ and understanding its reactivity with aliphatic hydroxyl groups will significantly broaden the scope of potential comonomers.

## Results and discussion

### Model studies

**Scheme 1:**
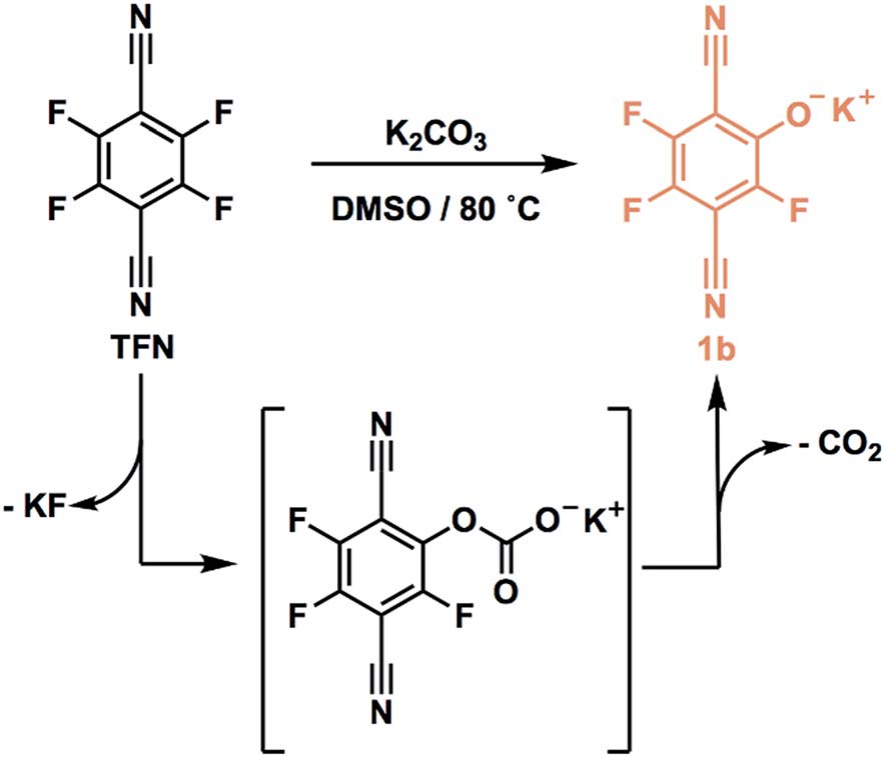


**TFN-CDP-1** was prepared in THF in 18% isolated yield under similar conditions as our previous report;^30^ it is synthesized *via* a S_N_Ar polycondensation in which the alcohol groups of β-CD are deprotonated by K_2_CO_3_. Yield is defined throughout this work as (mass of isolated TFN-CDP)/(mass of the monomers), because the theoretical yield depends on both the efficiency of monomer incorporation and the number of S_N_Ar reactions that occur. Furthermore, the ratio of TFN : β-CD (6 : 1) found in the isolated polymer deviated from the monomer feed ratio (3 : 1 TFN : β-CD). The low yield and difference between the monomer feed and incorporation ratios were suggestive of side reactions, which motivated us to study the reactivity of TFN. Polymerizations in DMSO (**TFN-CDP-2**) provided higher yields (64%) compared to **TFN-CDP-1**, even at shorter reaction times (18 h *vs.* 48 h). A monomer feed ratio of 6 : 1 TFN : β-CD provided an incorporation ratio of 5.2 : 1, corresponding to a loss of 0.8 equiv. (13 mol%) of the TFN. After isolating the insoluble polymer, analysis of the soluble fraction by ^19^F and ^13^C NMR spectroscopy and high-resolution mass spectrometry indicated the formation of phenolate **1b** as a major side product. We hypothesized that **1b** is formed *via* the S_N_Ar of TFN and K_2_CO_3_, followed by decarboxylation. Indeed, **1b** is formed quantitatively within 10 min when TFN (0.3 mM) and K_2_CO_3_ (3.3 equiv.) are combined in anhydrous DMSO at 80 °C (Scheme 1), as measured by ^19^F NMR spectroscopy. Evidence for this side reaction under the polymerization conditions, combined with the rapid formation of **1b** in the absence of other nucleophiles, suggested that further study of the phenolation of TFN might improve the yield of the polymerization.

**Scheme 2:**
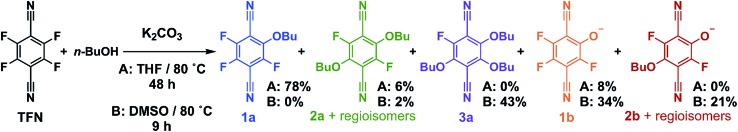


**Fig. 2:**
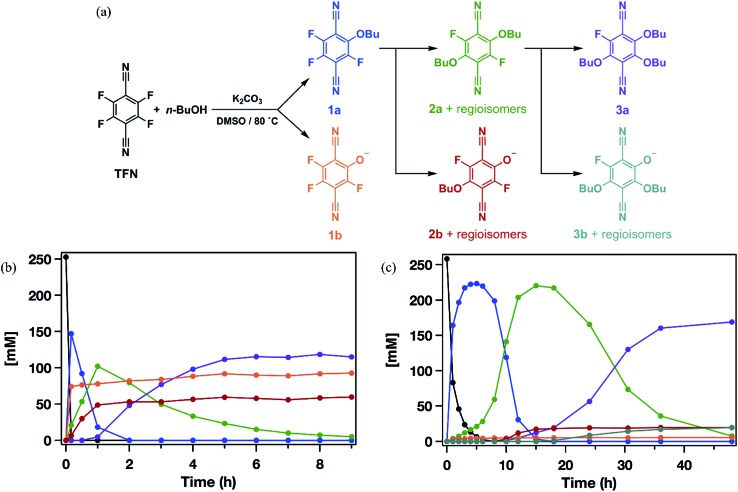
(a) Reaction pathways for the formation of *n*-butoxy and phenolate-substituted TFN derivatives. (b) Concentrations of each species as a function of reaction time observed when 3.3 equiv. K_2_CO_3_ is added at *t* = 0. (c) Concentrations of each species as a function of reaction time observed when 3.3 equiv. K_2_CO_3_ was added at 0.1 equiv. per h.

Model S_N_Ar reactions between TFN and *n*-butanol show that phenolation and etherification reactions are in competition. Etherification and phenolation both proceed slowly in THF over 48 h. The dominant product observed by ^19^F NMR spectroscopy was the monosubstituted ether **1a** (78%). Minor products included a mixture of dibutyl ethers **2a** (6%) and phenolate **1b** (8%). More highly substituted ethers and phenolate products were observed when the reaction was run in DMSO for 9 h. These higher substituted products may explain why the polymerization is both faster and higher yielding in DMSO. The major products (Scheme 2) were the trisubstituted ether **3a** (43%), **1b** (34%), a mixture of regioisomers containing one phenolate and one ether (**2b**, 21%), and a mixture of disubstituted ethers (**2a**, 2%). Phenolation is enhanced in this solvent, and **1b** is much less active for further substitutions as compared to the etherification products. When a pure sample of phenolate **1b** was subjected to these reaction conditions, only 4% conversion to the 1,2-substituted regioisomer of **2b** was observed after 24 h, indicating that **2b** forms *via***1a**. A model reaction in DMSO monitored by ^19^F NMR spectroscopy as a function of reaction time also demonstrates that phenolated products essentially do not undergo further reactions in the presence of competing electrophiles (Fig. 2a). Most of the TFN is consumed within 10 min, with 59% conversion to **1a** and 30% conversion to **1b**. **1a** continues to react to form more highly substituted products, whereas the concentration of **1b** remains approximately constant over 9 h. Most of **1a** reacts within 2 h to provide dibutyl ethers **2a** and phenolates **2b**, which also persist for the remainder of the reaction. **2a** reacts further to provide **3a** over the next several hours, and no evidence for the formation of dibutyl-ether/monophenolates (**3b**) was observed (Fig. 2b). These results provide significant insight into the formation of TFN-CD polymers. First, the competition between the first etherification and phenolation process in the first few minutes of the reaction are likely to influence yield and total incorporation of TFN derivatives in the resulting polymer since **1b** is mostly unreactive. Furthermore, the formation of **2b** demonstrates a process by which phenolate groups might be incorporated into the polymer.

The apparent competition between etherification and phenolation suggested that slow addition of the K_2_CO_3_ might suppress phenolation. A model reaction in DMSO in which K_2_CO_3_ was added gradually (3.3 equiv., 0.1 equiv. per h) provided sequential etherification from **1a** to **2a** to **3a** (Fig. 2c) as the dominant reaction pathway. After 6 h, 87% of the TFN was converted to **1a**, and only 2% proceeded to **1b**. This selectivity corresponds to a 94% reduction in phenolation relative to when K_2_CO_3_ is added at once. As before, **1a** reacts to form more substituted products, and the concentration of **1b** does not decrease at longer reaction times. After 15 h, the conversion to **2b** was only 7%, and the combined regioisomers of **2a**, represented 85% of the TFN-derived species. The concentration of the **2b** regioisomers also remain approximately constant for the remainder of the reaction, and **2a** proceeds to the trisubstituted product, **3a**, and the dibutoxy phenolate products, **3b**. After 48 h, **3a** made up 76% of the TFN-derived species, as compared to 43% when the base is present throughout the reaction. We attribute the formation of **3b** to the near-complete consumption of *n*-butanol at the later stages of the reaction. These experiments demonstrate that phenolation occurs readily in the presence of excess K_2_CO_3_ and that etherification is favored when the concentration of base is kept low. Therefore, the rate of K_2_CO_3_ addition might influence both the yield and degree of phenolate incorporation into TFN-CDP polymers.

### Polymer synthesis and characterization

**Scheme 3:**
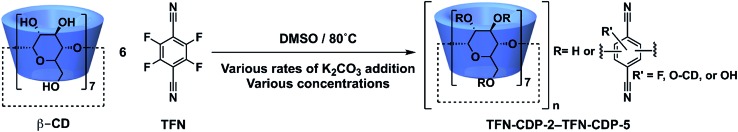


The above findings suggest that the K_2_CO_3_ addition rate will also affect the yield and properties of TFN-CDP (Scheme 3). For example, the formation of **1b** during the polymerization would decrease the yield as further substitution reactions on this species are slow. Later phenolation processes, analogous to the formation of **2b** or **3b**, do not decrease yield but incorporate phenolated TFN groups into the polymer network. We performed two polymerizations, either at [TFN] = 0.33 M and 0.17 equiv. β-CD, to which 3.3 equiv. K_2_CO_3_ were added all at once (**TFN-CDP-2**) or at 0.1 equiv. per h (**TFN-CDP-3**). The suppression of **1b** is reflected in the combustion analysis of the two polymers. The C : N ratio was used to determine the number of TFN per β-CD. **TFN-CDP-2** exhibits a TFN : β-CD ratio of 5.2, corresponding to a loss of 0.8 equiv. (13 mol%) TFN to **1b**. **TFN-CDP-3** had a TFN : β-CD ratio of 5.9, which indicates that only 0.1 equiv. (2 mol%) of the TFN was lost to the first phenolation process. **TFN-CDP-2** and **TFN-CDP-3** also had a different concentration of phenolate incorporated into the polymers (Table 1). The phenolate concentration in the polymers was determined by deprotonating the phenolates using Li_2_CO_3_ and determining the amount of bound Li ions using inductively coupled plasma optical emission spectroscopy (ICP-OES, see ESI† for detailed analysis procedures). To further validate this method, we performed a similar analysis of K^+^ content in the polymers by using K_2_CO_3_ in place of Li_2_CO_3_ and found similar phenolate loadings (Table S1†). **TFN-CDP-2** had a higher phenolate concentration (0.44 mmol g^–1^) compared to **TFN-CDP-3** (0.22 mmol g^–1^). These results are consistent with the model studies and demonstrate a means to control the phenolate concentration within the polymer networks.

**Table 1:** Polymerization conditions, bound phenolate concentrations, and TFN : CD ratio in polymer samples^*a*^

Polymer	[Phenolate] (mmol g^–1^)	K_2_CO_3_ addition rate	[TFN]_0_ (M)	TFN : CD ratio	*S*_BET_ (m^2^ g^–1^)	Yield (%)
**TFN-CDP-2**	0.44 (+/– 0.01)	At once	0.3	5.2	346 (+/– 113)	64
**TFN-CDP-3**	0.22 (+/– 0.02)	0.1 equiv. per h	0.3	5.9	Non-porous	67
**TFN-CDP-4**	0.14 (+/– <0.01)	At once	1.2	5.2	Non-porous	54
**TFN-CDP-5**	0.08 (+/– <0.01)	0.1 equiv. per h	1.2	5.9	Non-porous	54

^*a*^TFN-CDP refers to tetrafluoroterephthalonitrile-linked β-cyclodextrin polymer.

Further manipulation of the phenolate loading was achieved by using higher monomer concentration in the polymerization. TFN is no longer fully soluble under the reaction conditions at a concentration of 1.2 M. A TFN-CDP formulation was prepared (**TFN-CDP-4**) using a concentration of 1.2 M and by adding the K_2_CO_3_ all at once. **TFN-CDP-4** has fewer phenolates (0.14 mmol g^–1^) compared to **TFN-CDP-2** (0.44 mmol g^–1^), which is formed under similar conditions but lower initial monomer concentration. In the synthesis of **TFN-CDP-4**, the volume of DMSO was decreased four-fold, which renders a portion of the TFN undissolved and effectively increases the ratio of β-CD to dissolved TFN compared to reactions run at lower concentration. This higher effective ratio of β-CD alcohols to TFN-fluorides favors etherification over phenolation. Finally, combining both effects that provide lower phenolate concentration, **TFN-CDP-5** was synthesized using gradual K_2_CO_3_ addition (0.1 equiv. per h) and high concentration (1.2 M), which provided the lowest concentration of bound phenolate (0.08 mmol g^–1^) (Scheme 3). The results of these polymerizations demonstrate that CD-TFN polymers with varying phenolate concentrations can be accessed by exploiting the competition between etherification and phenolation.

### Pb^2+^ capacity

**Fig. 3:**
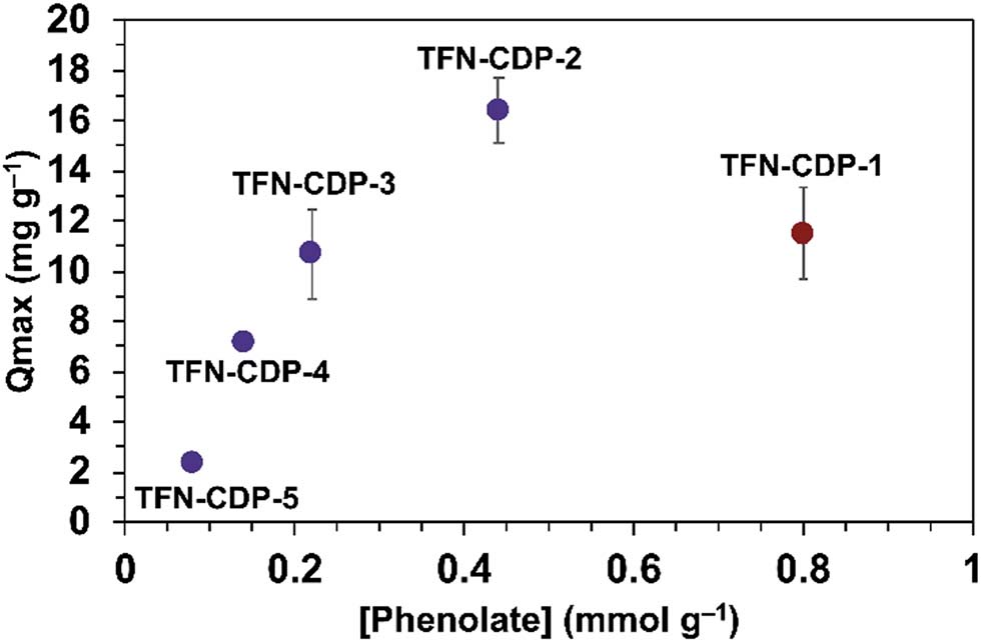
*Q*_max_ for Pb^2+^ as a function of bound phenolate functionalities in polymer samples **TFN-CDP-1–TFN-CDP-5**.

He and coworkers recently reported that a polymer synthesized under similar conditions as **TFN-CDP-1** binds Pb^2+^ ions with a high capacity (*Q*_max_ = 215 mg g^–1^).^37^ They attributed Pb^2+^ binding to interactions with the hydroxyl groups of β-CD, but the possible presence of phenolates in the polymers was not yet recognized. We evaluated TFN-CDP polymer samples of varying phenolate content to determine their role in sequestering Pb^2+^ ions. The *Q*_max_ of each polymer was determined by batch experiments in which the polymers were exposed to Pb(NO_3_)_2_ solutions (1–50 ppm, pH = 5) for 24 h. After removing the polymers by filtration, the Pb^2+^ concentrations were determined *via* ICP-OES. The Pb^2+^ removal was determined by the concentration difference between the polymer treated solution and control solutions not exposed to polymers. *Q*_max_ was determined by building an isotherm of the average solid phase concentration of Pb^2+^ verses the aqueous concentration of Pb^2+^ and fitting this to the Langmuir model. The *Q*_max_ for **TFN-CDP-1–TFN-CDP-5** was 16.4–2.4 mg g^–1^ (Fig. 3). Polymers prepared in DMSO (**TFN-CDP-2–TFN-CDP-5**) with varying phenolate content showed a strong positive correlation between Pb^2+^ capacity and phenolation, **TFN-CDP-1** was also found to have a high phenolate content and exhibited a reasonable capacity for Pb^2+^, but not as high as **TFN-CDP-2**. Unlike **TFN-CDP-2–TFN-CDP-5**, **TFN-CDP-1** was synthesized under different conditions (THF) under which β-CD is insoluble. Therefore, it may have other structural differences, such as variations in local TFN functionalization density or regioselectivity in its reactions with β-CD that also influence its Pb^2+^ binding. We have not explored these differences further because **TFN-CDP-1** is difficult to scale up and shows lower Pb^2+^ binding capacity than **TFN-CDP-2.** Although the *S*_BET_ of the materials are different, it is likely that the polymer is fully accessible to pollutants at equilibrium. This point is consistent with bisphenol-A (BPA) isotherms generated for each of the polymers (Fig. S4†), from which Langmuir fits provided high *Q*_max_ (>1 BPA : β-CD) for both porous and non-porous polymers. Therefore, the differences in *Q*_max_ for Pb^2+^ binding likely arise from the variation in phenolate loading of each polymer. Finally, it is important to note that all of our polymers show approximately 20 fold inferior capacity to the very high value (215 mg g^–1^) reported by He and coworkers^37^ for a polymer prepared similarly to **TFN-CDP-1**. The discrepancy between our measured values and this report cannot be readily explained.

The rate of Pb^2+^ removal is dependent on the porosity in these polymers. To probe the importance of porosity to Pb^2+^ removal, the rate of Pb^2+^ removal (Pb^2+^*k*_obs_) was determined for all polymers (Fig. S6†). **TFN-CDP-2** has a higher surface area than non-porous **TFN-CDP-3–TFN-CDP-5** and has superior Pb^2+^*k*_obs_ compared to the other polymers. Among the non-porous polymers, the phenolate content did not have a pronounced effect on Pb^2+^*k*_obs_ but did have an effect on the capacity at equilibrium (*q*_e_). These results further suggest that phenolate concentration is correlated with affinity to Pb^2+^ ions at low concentrations.

### Micropollutant affinity

**Fig. 4:**
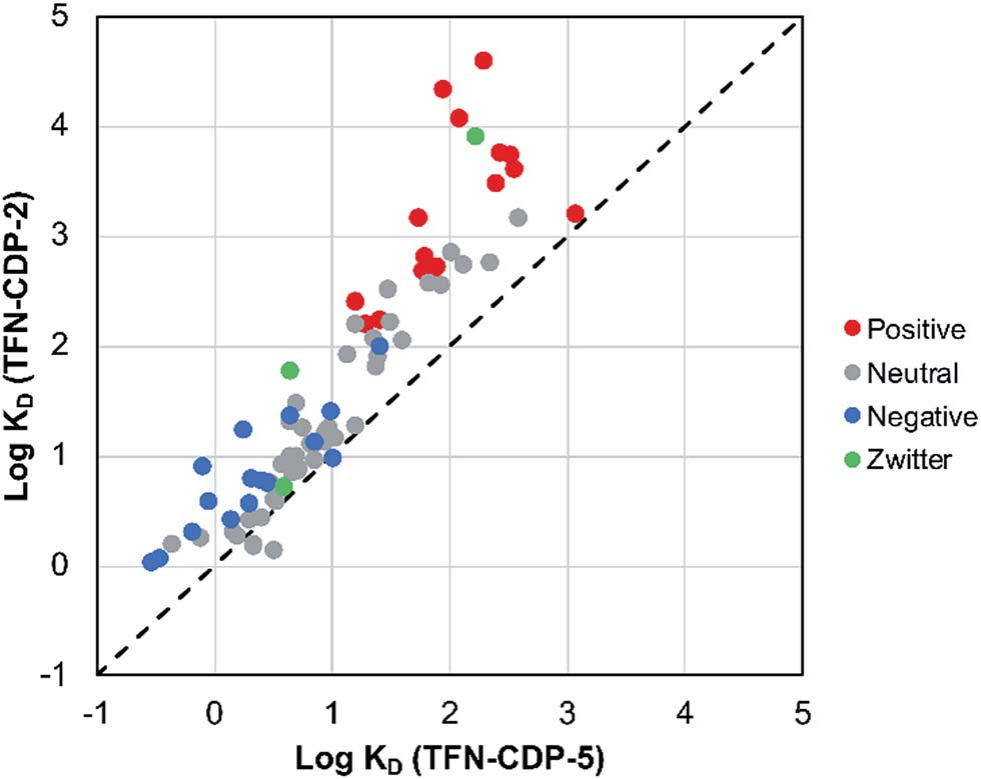
Affinity experiment comparing higher-phenolate-content **TFN-CDP-2** and lower-phenolate-content **TFN-CDP-5**. This experiment shows that **TFN-CDP-2** has higher affinities for 81 of 83 micropollutants tested.

MP affinity testing on all polymer samples indicate that phenolation is desirable in that these charged groups in the polymer predictably affect polymer affinity to positively charged MPs. MP affinity was determined by measuring the adsorption of a mixture of 83 MPs at low concentrations (1 μg L^–1^; 1 ppb) by means of HPLC-MS/MS (Table S2†). The adsorption was used to determine the affinity of each MP (*K*_D_) for each polymer. To compare the MP affinity of highly phenolated **TFN-CDP-2** with weakly phenolated **TFN-CDP-5** the log *K*_D_ for each MP for the two polymer samples was plotted on the binary chart in Fig. 4. Of the 83 micropollutants, 81 bind more strongly to the more heavily phenolated polymer. Not surprisingly, cationic substances bind with the highest *K*_D_ and show the largest differential affinity for the more heavily phenolated polymer. However, although anionic substances bind with lower affinity, they still bind to the more heavily phenolated polymer **TFN-CDP-2** more strongly than **TFN-CDP-5**. Similar affinity trends for both cationic and anionic substances were observed in pairwise comparisons of the polymers with intermediate phenolate content (Fig. S3†). These findings indicate that TFN phenolation is correlated with improved affinity for cationic MPs yet has a smaller and often non-deleterious impact on binding of anionic and uncharged substances.

The phenolate content of the non-porous samples (**TFN-CDP-3** through **TFN-CDP-5**) also influences their rates of MP removal. To probe this effect, the pseudo 2^nd^-order rate constant for BPA removal (BPA *k*_obs_) was determined for all polymers under identical conditions (Fig. S5†). Among these non-porous samples, the BPA *k*_obs_ is correlated to phenolate concentration, even though BPA is a neutral molecule. We speculate that the phenolated polymers are more hydrophilic, which lead to faster diffusion of BPA-contaminated water into the polymer network. In addition, the differences in *S*_BET_ also affect BPA *k*_obs_. Porous **TFN-CDP-2** has a significantly higher surface area than non-porous **TFN-CDP-3** through **TFN-CDP-5** and has a BPA *k*_obs_ 1–2 orders of magnitude higher than the other polymers (1.61 and 0.02–0.23 g mg^–1^ min^–1^, respectively). These experiments demonstrate that both phenolation and porosity affect the rate of BPA removal.

## Conclusions

An improved understanding of the S_N_Ar polycondensation of aliphatic alcohols and TFN was achieved using model etherification reactions, the first in depth such studies for TFN and aliphatic alcohols. These findings were adapted to synthesize TFN-CDPs with varying phenolate contents. The results show that the main factors in controlling the inclusion of phenolic functional groups are concentration of the reaction and rate of base addition. Polymers with higher concentrations of phenolic functionalities had higher affinity for 81 MPs, particularly those that are positively charged, as well as higher Pb^2+^ binding capacity. These results suggest that high phenolate content in TFN-CDP is key for Pb^2+^ ion capacity and MP affinity. Finally, this study demonstrates that the rational synthesis of TFN-CDPs can be achieved by controlling the rate of K_2_CO_3_ addition and the concentration of reactants. Future studies will focus on the incorporation of chelating groups to increase the capacity of CDPs for lead ion binding and expand the application of CDPs to the removal of other heavy metal ions.

## Conflicts of interest

D. E. H. and W. R. D. serve on the scientific advisory board and own equity and/or stock options in CycloPure, Inc., which is commercializing related cyclodextrin polymers.

## Supplementary Material

Supplementary information
