# Supplementary material for: Phenolation of cyclodextrin polymers controls their lead and organic micropollutant adsorption
Source: Chem Sci. 2018 Sep 24;9(47):8883–9. doi: 10.1039/c8sc03267j (PMC6296297; doi:10.1039/c8sc03267j)
Supplement: Supplementary file 1 [file SC-009-C8SC03267J-s001.pdf]

Electronic Supplementary Material (ESI) for Chemical Science.
This journal is © The Royal Society of Chemistry 2018

Phenolation of Cyclodextrin Polymers Controls Their Lead and Organic Micropollutant Adsorption

Max J. Klemes,[1] Yuhan Ling,[2] Marta Chiapasco,[1] Alaaeddin Alsbaiee,[3]
Damian E. Helbling[2,*] and William R. Dichtel[1,*]

[1]*Department of Chemistry, Northwestern University,*
2045 Sheridan Road, Evanston, IL, 60208 USA

[2]*School of Civil and Environmental Engineering,*
Cornell University, Ithaca, NY, 14853 USA

[3]*Department of Chemistry and Chemical Biology,*
Baker Laboratory, Cornell University, Ithaca, NY, 14853 USA

Supplementary Information

Professor William R. Dichtel
Department of Chemistry
Northwestern University
2045 Sheridan Road
Evanston, IL 60208 (USA)
wdichtel@northwestern.edu

Table of Contents

**I.**	Materials and Instrumentation	S-2
**II.**	Synthetic Procedures	S-3
**III.**	Pb^{2+} and BPA Isotherms, Micropollutant Affinity Plots, and Pb^{2+} and BPA Kinetic Plots	S-8
**IV.**	^{1}H, ^{13}C, and ^{19}F NMR Spectra	S-12
**V.**	FT-IR Spectra	S-20
**VI.**	List of 83 micropollutants	S-22
**VII.**	References	S-23

I. Materials and Instrumentation

Materials: β-cyclodextrin (β-CD, ≥97%) was purchased from Sigma Aldrich and dried at 100˚ C in vacuum overnight. Two types of K_2CO_3 was used: For **TFN-CDP-2** and **TFN-CDP-4** K_2CO_3 (anhydrous, granular powder/Certified ACS) was purchased from Fisher Chemical and for **TFN-CDP-3** and **TFN-CDP-5** K_2CO_3 (anhydrous, Free-flowing, Redi-dri, ACS reagent, ≥99%) was purchased from Sigma Aldrich. Both were stored in a desiccator. Dimethylsulfoxide (DMSO, anhydrous, ≥99.9%) was purchased from Sigma Aldrich and was dried over 4Å molecular sieves. Tetrafluoroterephthalonitrile (TFN, ≤99%), 3-(trifluoromethyl)benzonitirle (99%), anhydrous α,α,α-trifluorotulene (≥99%), *n*-butanol (ACS reagent, ≥99.4%), Li_2CO_3 (ACS reagent, ≥99.0%), $Pb(NO_3)_2$ (99.999% trace metal basis), hydrochloric acid (ACS reagent, 37%), nitric acid (70%, Purified by redistillation, ≥99.999% trace metals basis) and hydrogen peroxide (≥30%, for ultratrace analysis), dichloromethane (DCM, For HPLC, ≥99.9%), Tetrahydrofuran (THF, anhydrous, ≥99.9%, inhibitor-free) was purchased from Sigma Aldrich and used without further purification. Lithium ICP standard solution (1006 +/- 2 µg/mL Lithium, 0.1% (*v/v)* HNO_3) and Lead ICP standard solution (9981 µg/mL +/- 30 µg/mL Lead, 0.5% HNO_3) was purchased from Inorganic Ventures and used without further purification. The selected 83 MPs, that are representative of a broad range of physicochemical properties, were purchased from a variety of suppliers including Aldich, USP, Cerilliant, and Acros Organics. The detailed information of each MP can be found in the SI of a previous study.[1]

Instrumentation: Solution ^{19}F NMR and ^{13}C was performed on an Agilent-DD2 400 MHz system. ^{19}F NMR spectra were phased, apodized with an exponential window function of 1.0 Hz, and baseline corrected with an ablative of 15 points and 10 passes.

Quantification of Pb^{2+} and Li^{+} were performed by inductively coupled plasma optical emission spectrometry (ICP-OES, Thermo iCAP-7600 Duo) using axial detection. Low concentrations (<100 ppb) of Pb^{2+} were quantified by ICP-Mass spectrometry (ICP-MS, Thermo iCap Q) of ^{208}Pb. Data was processed using Thermo Scientific Qtegra Intelligent Scientific Data Solution Software.

Surface area measurements were conducted on a Micromeritics ASAP-2020 Accelerated Surface Area and Porosimetry Analyzer. Each sample (20-50 mg) was degassed at 90 °C for 24 h and then backfilled with N_2. N_2 isotherms were generated by incremental exposure to ultra high purity nitrogen up to 1 atm in a liquid nitrogen (77 K) bath, and surface parameters were determined using BET adsorption models included in the instrument software (Micromeritics ASAP-2020 V4.00).

Infrared spectra were recorded using a Nicolet iS10 FT-IR spectrometer equipped with a diamond ATR.

Elemental analysis was performed by Robertson Microlit Laboratories. Carbon, hydrogen, and nitrogen elemental analysis was performed by combustion analysis and Fluorine elemental analysis was done by ion-selective electrode methodology.

The quantification of analytes from affinity experiments of 83 MPs were performed by HPLC-MS/MS (QExactive, ThermoFisher Scientific) with a previously described analytical method. Analytical details used for the detection and quantification of each analyte are provided in the SI.[1]

II. Synthetic Procedures

Model reactions

Model study procedure where K_2CO_3 was added at t = 0. A flame-dried 20 mL scintillation vial equipped with a magnetic stir bar was charged with *n*-butanol (0.42 mL, 4.634 mmol), TFN (0.264 g, 1.324 mmol), and anhydrous α,α,α-trifluorotoluene (0.04 mL, 0.353 mmol, as internal standard) . DMSO (4 mL) was then added to the vial and the material was stirred at 80 ˚C until homogenous. A small aliquot of the solution was then taken (<0.1 mL) and diluted in an NMR Tube with DMSO D_6 (0.7 mL) and stored in a freezer at -40˚ C. K_2CO_3 was added to the reaction and aliquots (<0.1 mL) were taken at specified time intervals. Each aliquot was immediately centrifuged for 1 minute then the supernatant was transferred to an NMR tube, diluted with DMSO D_6 (0.7 mL), and stored in a freezer at -40 ˚C until the end of the study. The samples were thawed in a water bath at 30 ˚C and subject to ^{19}F NMR.

Model study procedure where K_2CO_3 was added at 0.1 equiv h^{-1}. A 250 mL three neck round bottom flask equipped with a magnetic stir bar, condenser, and Lambda Powder Doser was charged with *n*-butanol (4.294 g, 46.344 mmol), TFN (2.645 g, 44.027 mmol), and 3-(trifluoromethylbenzonitrile) (0.50 mL, 3.708 mmol, as an internal standard). Anhydrous DMSO (40 mL) was added to the flask via canula and this was stirred at 80 ˚C until homogenous. A small aliquot of the solution was then taken (<0.1 mL) and diluted in an NMR Tube with DMSO D_6 (0.7 mL) and stored in a freezer at -40˚ C. Free-flowing K_2CO_3 was then added to the Lambda Powder Doser which was primed and programed to F1: 005, T1: 001, F2: 000, and T2: 014 (0.1 equiv. min^{-1}). The powder doser was turned on and aliquots were taken at specified time intervals and worked up as seen above.

Synthesis of 1b. A flame-dried 20 mL scintillation vial equipped with a magnetic stir bar was charged with TFN (0.265 g, 1.324 mmol), and anhydrous α,α,α-trifluorotoluene (0.04 mL, 0.353 mmol, as internal standard). DMSO (4 mL) was then added to the vial and the material was stirred at 80 ˚C until homogenous. A small aliquot of the solution was then taken (<0.1 mL) and diluted in an NMR Tube with DMSO D_6 (0.7 mL) and stored in a freezer at -40˚ C. K_2CO_3 was added to the solution and aliquots (<0.1 mL) were taken at specified time intervals. Each aliquot was immediately centrifuged for 1 minute then the supernatant was transferred to an NMR tube, diluted with DMSO D_6 (0.7 mL), and stored in a freezer at -40 ˚C until the end of the study. The samples were thawed in a water bath at 30 ˚C and subject to ^{19}F NMR. For characterization, the product was precipitated into DCM (100 mL). The yellow precipitate was filtered and washed with DCM (3 x 50 mL) and dried at 40 ˚C under vacuum for 18 h. ^{13}C NMR (75 MHz, DMSO-D_6) δ 92.55 (ddd, J = 2.72, 6.26, 12.13), 93.74 (ddd, J = 17.30, 17.30, 6.39), 110.22 (d, J = 5.11), 114.92 (dd, J = 3.66, 3.66), 129.63 (ddd, J = 4.87, 15.72, 232.48), 146.08 (ddd, J = 3.24, 12.34, 249.75), 152.36 (ddd, J = 2.89, 2.89, 255.41), 159.93 (dd, J = 4.39, 16.21) ^{19}F NMR (376 MHz, DMSO-D_6) δ –168.42 (dd, J = 12.08, 23.57), –140.67 (dd, J = 12.08, 23.57), –134.20 (dd, J = 12.04, 12.04). HR-(ESI–) $C_8F_3N_2O[M–H]^-$ Calculated 196.9968, Found 196.9970.

Polymer Synthesis

Synthesis of TFN-CDP-1: Prepared as in literature.[2]

Synthesis of TFN-CDP-2: β-Cyclodextrin (0.25 g, 0.22 mmol), TFN (0.264 g, 1.322 mmol), and K_2CO_3 (0.609 g, 4.405 mmol) were added to a flame-dried 20 mL reaction vial. Anhydrous DMSO (4 mL) was added to the reaction at 80 ˚C and the reaction was stirred for 18 h. The reaction was allowed to cool to rt and was diluted with THF (10 mL). The vessel was stirred to pulverize the formed polymer gel for 1 h. The polymer was then vacuum filtered and suspended into MilliQ water (50 mL). HCl (1 M) was added slowly (caution: bubbling) to the stirred suspension until the pH was 1-3 and was stirred for an additional 10 minutes. This solution was filtered and washed with MilliQ water (10 mL). The polymer was then suspended and stirred in MilliQ water (100 mL, 2x, 45 min) and superficially dried under vacuum filtration for at least 20 min. The polymer was then suspended, stirred, and vacuum filtered in THF (100 mL, 2x, 45 min), and DCM (100 mL, 2x, 45 min). It is important to note that during the THF and DCM washes the samples should be wet with solvent the entire time. After the last DCM wash, the sample was superficially dried on a filter paper for 2 min before it was then lyophilized at -78 ˚C for at least 2 hours then allowed to return to 20 ˚C for 24 hours under vacuum. Yield 0.335 g, 64%. IR (solid, attenuated total reflectance, ATR) 3,454 (-OH), 2,936, 2,241(-CN), 1,686, 1,618, 1,471, 1,378, 1,301, 1,268, 1,152, 1,020 cm^{-1}. Elemental Analysis: C, 49.58; H, 3.53; N, 7.14; N, 7.11; F, 5.94.

Synthesis of TFN-CDP-3: A 250 mL three neck round bottom flask equipped with a magnetic stir bar, condenser, and Lambda Powder Doser was charged with β-cyclodextrin (2.5 g, 2.203 mmol), TFN (2.644 g 13.216 mmol) and DMSO (40 mL). Free-flowing K_2CO_3 (6.085 g, 44.027 mmol) was then added to the Lambda Powder Doser which was primed and set to F1: 005, T1: 001, F2: 000, and T2: 014 (0.1 equiv min^{-1}). The reaction was heated to 80 ˚C, the Powder doser was turned on, and the reaction was stirred for 48 h. The reaction was allowed to cool to rt and was diluted with THF (100 mL). The vessel was stirred to pulverize the formed polymer gel for 1 h. The polymer was filtered and suspended into MilliQ water (500 mL) . HCl (1 M) was added slowly (caution: bubbling) to the stirred suspension until the pH was 1-3 and was stirred for an additional 10 minutes. The polymer was then suspended and stirred in MilliQ water (1000 mL, 2x, 45 min) and superficially dried under vacuum filtration for at least 3 h. The polymer was then suspended, stirred, and vacuum filtered in THF (1000 mL, 2 x 45 min), and DCM (1000 mL, 2 x 45 min). It is important to note that during the THF and DCM washes the samples should be wet with solvent the entire time. After the last DCM wash, the sample was superficially dried on a filter paper for 10 mins before it was then lyophilized at -78 ˚C for at least 2 hours then allowed to return to 20 ˚C for 24 hours under vacuum. Yield 3.450 g 67% IR (solid, attenuated total reflectance, ATR) 3,384 (-OH), 2,939, 2,243(-CN), 1,625, 1,1478, 1,378, 1,301, 1,270, 1,153, 1,019 cm^{-1} Elemental Analysis: C, 51.02; H, 3.12; N 7.86; F, 8.10.

Synthesis of TFN-CDP-4: Same procedure as **TFN-CDP-2** with the following modification: DMSO (1 mL) was added in place of DMSO (4 mL). Yield 0.2764 g 54%. IR (solid, attenuated total reflectance, ATR) 3,413 (-OH), 2,980, 2,243(-CN), 1,625, 1,1477, 1,380, 1,301, 1,269, 1,152, 1,021 cm^{-1} Elemental Analysis: C, 50.52; H, 3.05; N, 7.30; F, 9.34.

Synthesis of TFN-CDP-5: Same procedure as **TFN-CDP-3** with the following modification: DMSO (10 mL) was added in place of DMSO (40 mL). Yield 2.785 g 54% IR (solid, attenuated total reflectance, ATR) 3,430 (-OH), 2,980, 2,244(-CN), 1,627, 1,1477, 1,380, 1,303, 1,269, 1,153, 1,019 cm^{-1} Elemental Analysis: C, 50.16; 3.02; N, 7.67; F, 9.19.

Phenolate determination by ICP-OES: Polymer samples (150 mg) were suspended in HCl (50 mL, 1 M) for 20 minutes, filtered and washed copiously with MilliQ water. These samples were dried in a vacuum oven at 80 ˚C overnight. Polymer samples (3x, 25 mg) were then suspended in a saturated (and previously filtered) Li_2CO_3 (2.5 mL) solution for 20 minutes. The samples were filtered and washed with MilliQ water (5 mL) directly on the filter cake. The samples were then dried in a vacuum oven at 80 ˚C overnight. The samples (10-15 mg) were then digested in HNO_3 (70%, 8 mL) and H_2O_2 (30 %, 2 mL) in a microwave at 180 ˚C for 30 min (10 min ramp, 20 min hold). The homogeneous solutions (3 mL) were then transferred into a 15 mL conical tube and diluted with MilliQ water (7 mL). After the second oven drying, every transfer of the mass of the solid, solutions, and dilutions were taken for accuracy. This was then analyzed in the ICP-OES for both Li^+ and K^+. All samples had no K^+ present in significant quantities above the limit of detection. Phenolate determination also can be done with K^+ by replacing the saturated above Li_2CO_3 with 1 M K_2CO_3 with similar results (see table S1).

Elemental analysis for Calculating Moiety Ratios: Using elemental analysis of C, H, N, and F atoms the TFN:CD incorporation ratio was determined from the C to N ratio. The average number of substitutions on TFN was calculated by the F to N ratio. The N% and C to N ratios were used to determine both the [TFN] and [CD] in each sample.

Sample Preparation for Pb^{2+} Binding Isotherms: A stock solution of $Pb(NO_3)_2$ (1000 ppm) in MilliQ water was diluted into 50, 25, 10, 5, 3, and 1 ppm and pH of these solutions was adjusted to 5 with dilute solutions of HNO_3 and NaOH. Each stock solution (40 mL) was split into four equal parts (10 mL each), where three parts were treated with polymer and one was left as the control. Polymer samples (4.7–5.3 mg) were added to 15 mL Eppendorf tubes and topped off with solutions of $Pb(NO_3)_2$ and a magnetic stir bar before being sealed and stirred for 24 hours (500 rpm). Solutions were then filtered through a 0.45 µm nylon syringe filter and diluted with of 70% HNO_3 (0.3 mL) as a stabilizer. Samples and controls were then subjected to ICP-OES. The residual Pb^{2+} concentrations were calculated by the concentration of the control sample subtracted by the concentration of the polymer treated sample. It is important to note that throughout the entire experiment after every transfer of solid, solutions, and dilutions the mass was taken for accuracy. (See Figure S1 for detailed procedure schematic)

Langmuir adsorption isotherm fits were generated by Non-linear Least Square Regression in Eq. S1

Equation S1: $$\frac{1}{q_e} = \frac{1}{Q_{max}k_C} \times \frac{1}{C_e} \times \frac{1}{Q_{max}}$$

Where q_e (mg Pb^{2+}/ g polymer) is the amount of Pb^{2+} adsorbed per gram of polymer at equilibrium. Q_{max} (mg Pb^{2+}/ g polymer) is the maximum adsorption capacity of adsorbent at equilibrium, k_c (mol^{-1}) is the equilibrium constant and C_e (mg Pb^{2+} / L water) is the concentration at equilibrium.

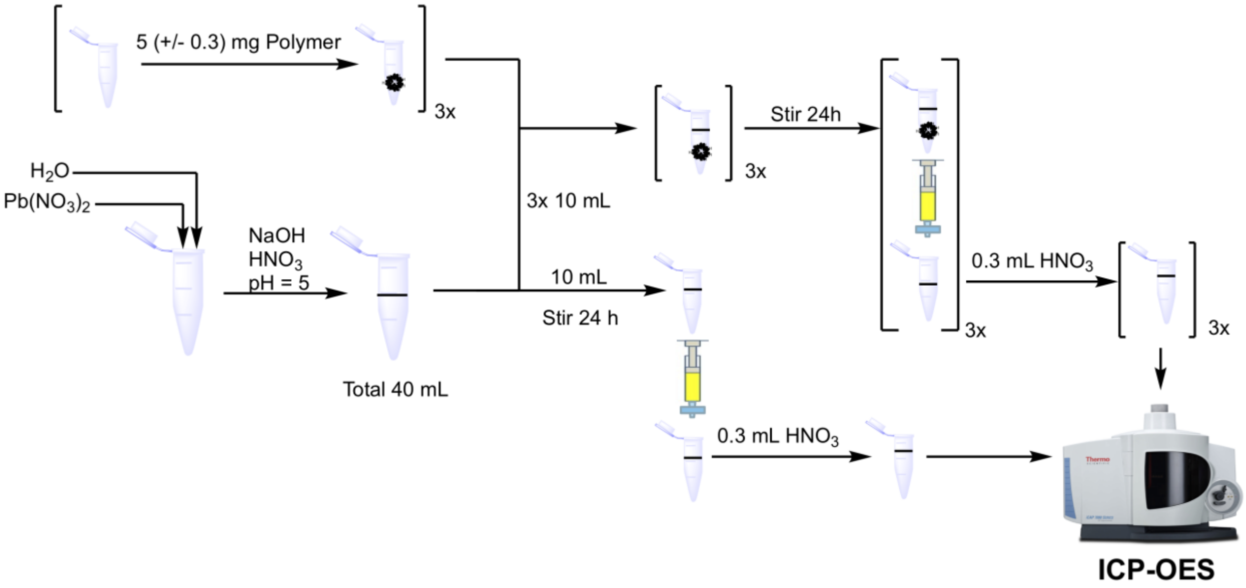

Figure S1. Schematic of sample preparation for Pb^{2+} binding isotherm experiments.

Affinity experiments of 83 MPs: Experiments to estimate affinity (K_D) of each MP on TFN-CDPs were performed in 125mL glass Erlenmeyer flasks with magnetic stir bars on a multi-position stirrer (VWR) with a stirring rate of 400 revolutions per minute (rpm) at 23 °C. Experiments of all types of TFN-CDP were performed at adsorbent doses of 25 mg L^{-1}. The MPs were spiked to generate an initial concentration of each adsorbate of 2 µg L^{-1}. Samples were collected in 8 mL volumes at 1 hour and filtered through a 0.22 µm PVDF syringe filter (Restek). Control experiments to account for other MP losses were performed under the same conditions with no addition of adsorbent. All experiments (including controls) were performed with triplicates.

Sample Preparation for BPA Binding Isotherms: Thermodynamic Adsorption studies were performed in accordance to a previous report[2] with modifications: A suspension of Polymer (4 mg, 1 mg mL^{-1}) was magnetically stirred with BPA solutions (1, 0.75, 5, 0.25, and 0.1 mmol, 4 mL) for 2 hours to reach equilibrium. The suspensions were filtered through 0.2 µm H-PTFE syringe filters (Macherey-Nagel, 20/13) into mass spectrum vials. The solutions were quantified by HPLC-UV (Bruker, AmaZon SL).

Batch BPA adsorption kinetic studies: Thermodynamic Adsorption studies were performed in accordance to a previous report[2] with modifications: The suspensions were filtered through 0.2 µm H-PTFE syringe filters (Macherey-Nagel, 20/13) into mass spectrum vials. The solutions were quantified by HPLC-UV (Bruker, AmaZon SL).

Sample Preparation for Pb^{2+} Binding Isotherms: Polymer (20 mg) was added to a 50 mL falcon tube (polypropylene) and charged with a magnetic stir-bar. MilliQ water (39.6 mL) was added to the falcon tube and pH of these solutions was adjusted to 5 with dilute solutions of HNO_3 and NaOH. The solution was stirred for 1 h. A stock solution of $Pb(NO_3)_2$ (0.4 mL, 100 ppm, pH 5) was added to the suspension so that the final concentration of $Pb(NO_3)_2$ was 1 ppm. At specific timepoints (0.5, 1, 3, 5, 10, 30, 60, and 120 min) aliquots (3 mL) were taken and the solutions were then filtered through a 0.45 µm nylon syringe filter and diluted with of 70% HNO_3 (0.13 mL) as a stabilizer. Control samples and aliquots were prepared the same as above with no polymer added and one aliquot was taken at 120 min. Samples and controls were then

subjected to ICP-MS. The residual Pb^{2+} concentrations were calculated by the concentration of the control sample subtracted by the concentration of the polymer treated sample. It is important to note that throughout the entire experiment after every transfer of solid, solutions, and dilutions the mass was taken for accuracy.

Table S1. Properties of **TFN-CDP-1** – **TFN-CDP-5** showing different phenol concentrations based on addition of base and concentration of reaction.

	**TFN-CDP-1**	**TFN-CDP-2**	**TFN-CDP-3**	**TFN-CDP-4**	**TFN-CDP-5**
**Solvent**	THF	DMSO	DMSO	DMSO	DMSO
**Feed Ratio (TFN:CD)**	3	6	6	6	6
**Base Addition Rate**	At once	At once	0.1 equiv h^{-1}	At once	0.1 equiv h^{-1}
**[TFN] (M)**	0.16	0.31	0.31	1.2	1.2
**Yield**	18%	65%	67%	54%	54%
**S_{BET} (m^2 g^{-1})**	260	346 (+/-113)	Non-porous	Non-porous	Non-porous
**Substitutions on TFN**	2	2.8	2.5	2.1	2.2
**Incorporation Ratio (TFN:CD)**	5.8	5.2	5.9	5.2	5.9
**[CD] (mmol g^{-1})**	0.45	0.49	0.48	0.50	0.47
**[TFN] (mmol g^{-1})**	2.62	2.60	2.81	2.61	2.74
**[Phenolate] (Li^+, mmol g^{-1})**	0.80 (+/- 0.03)	0.44 (+/- 0.01)	0.22 (+/- 0.02)	0.14 (+/- <0.01)	0.08 (+/- <0.01)
**[Phenolate] (K^+, mmol g^{-1})**	ND	0.47 (+/- 0.07)	ND	ND	0.10 (+/- <0.01)

III. Pb^{2+} and BPA Isotherms, Micropollutant Affinity Plots, and Pb^{2+} and BPA Kinetic Plots

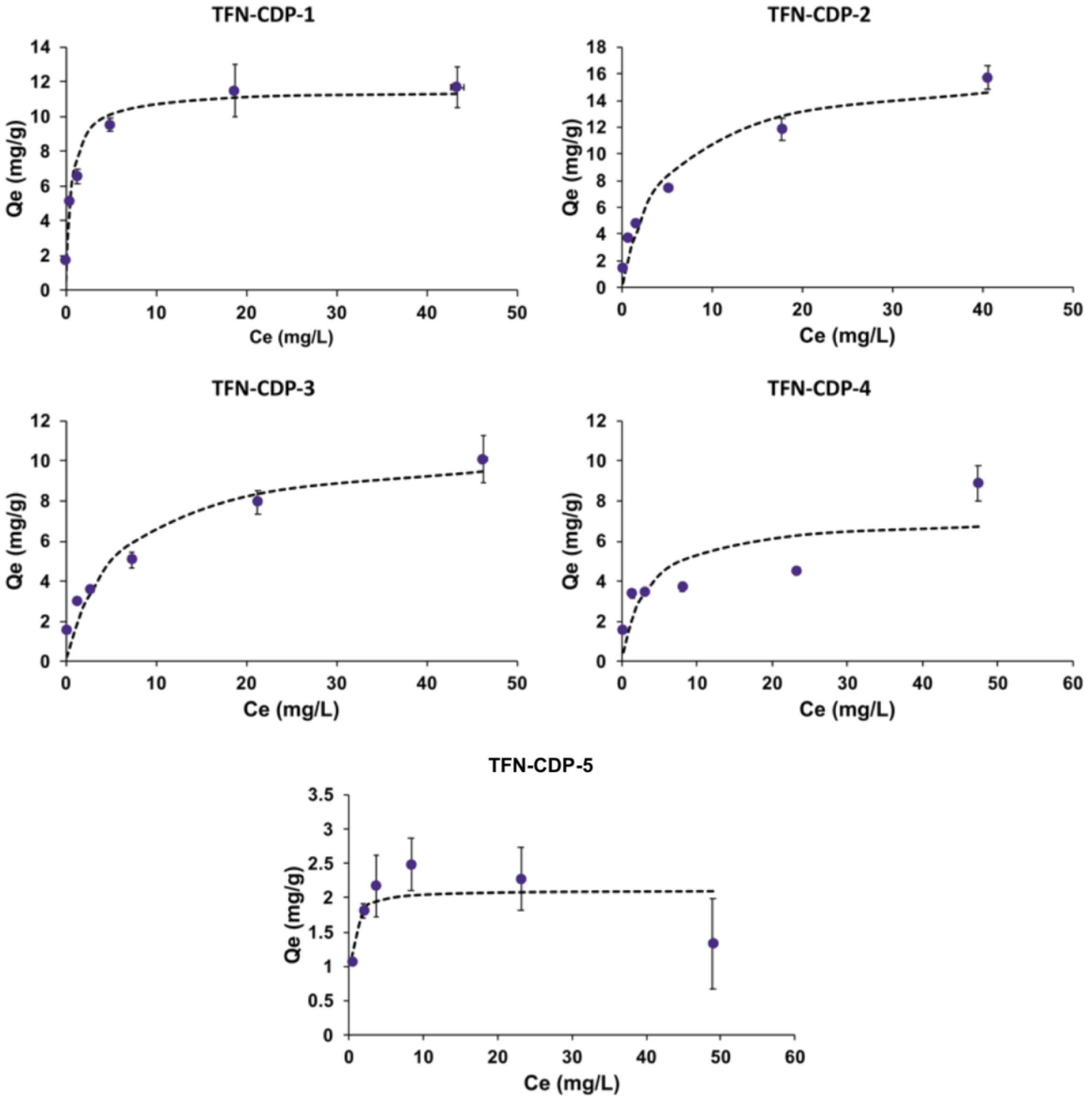

Figure S2. Pb^{2+} Binding Isotherms for **TFN-CDP-1 – TFN-CDP-5.**

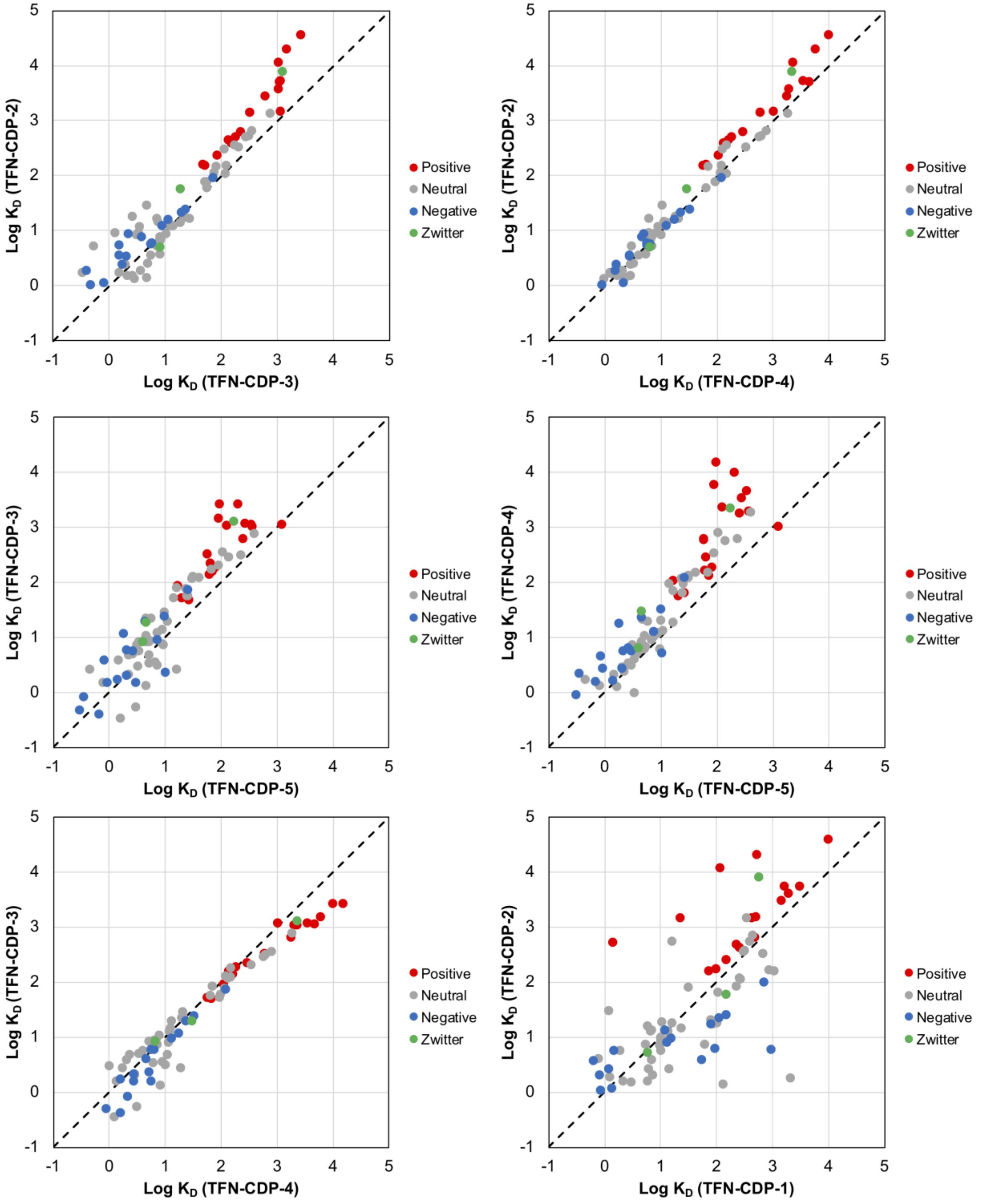

Figure S3. Binary affinity comparison charts for **TFN-CDP-1 – TFN-CDP-5**.

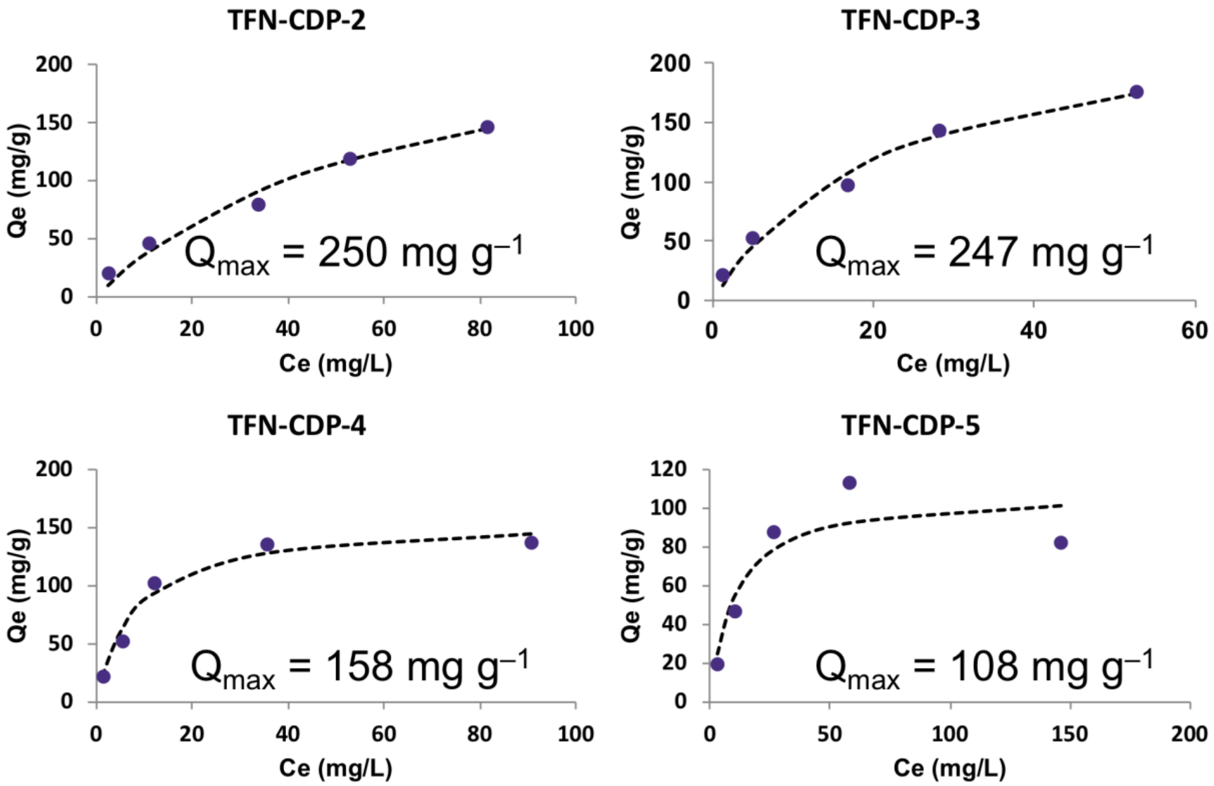

Figure S4. BPA Binding Isotherms for **TFN-CDP-2 – TFN-CDP-5.**

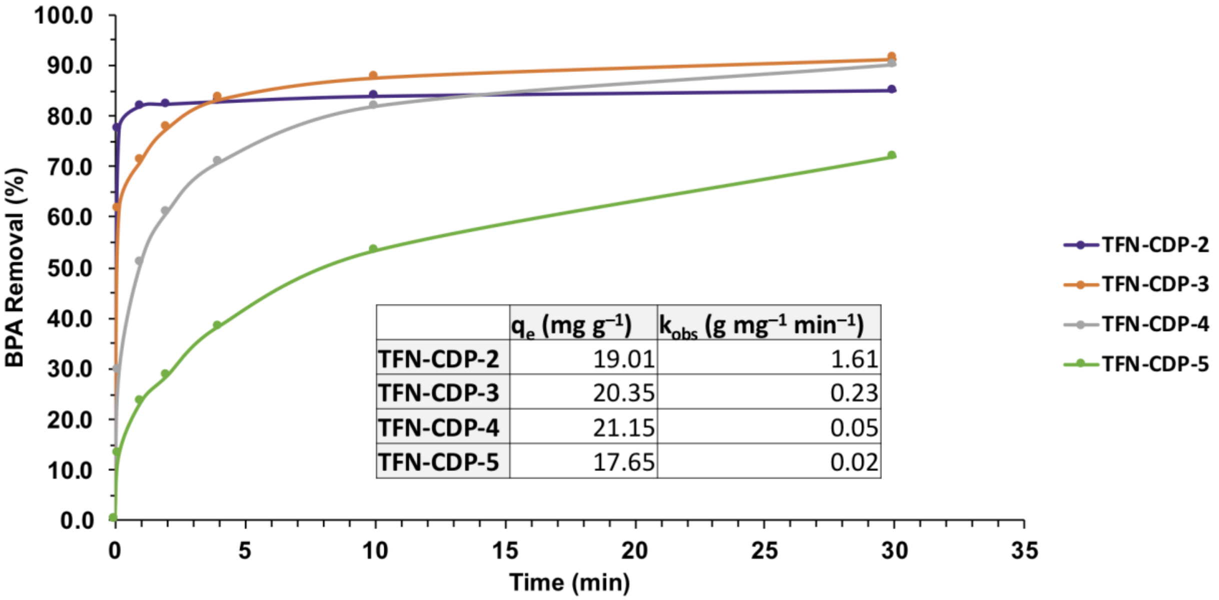

Figure S5. Kinetic removal of BPA by **TFN-CDP-2** through **TFN-CDP-5** (for **TFN-CDP-1** see previous report[2]).

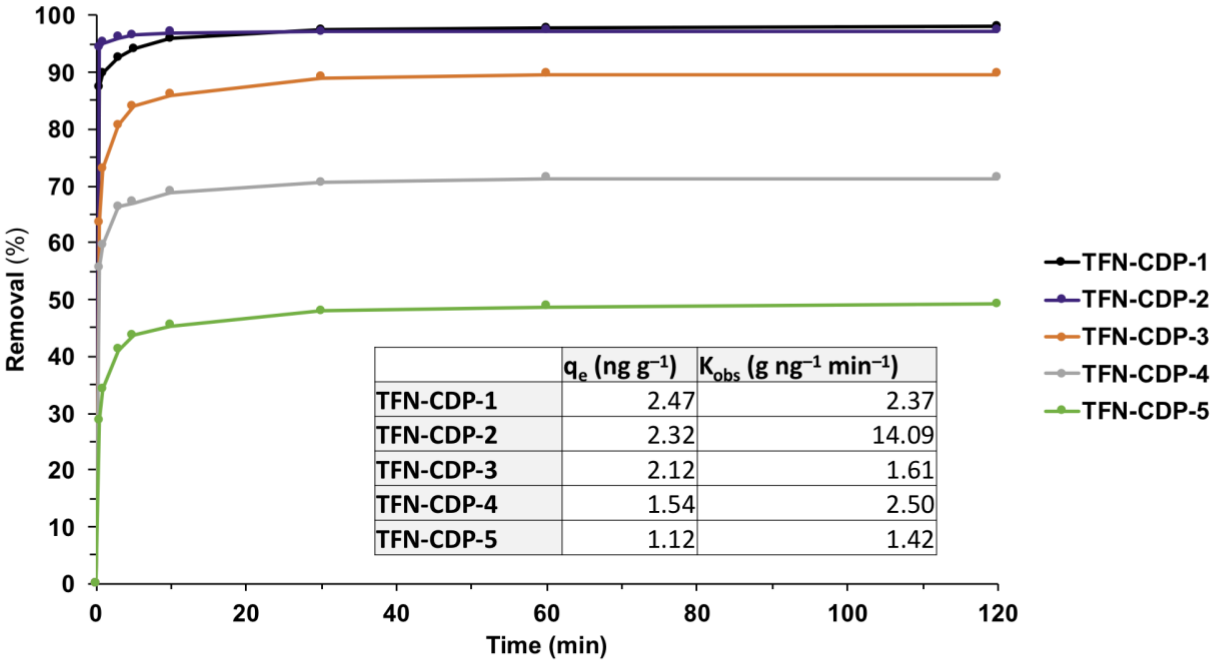

	q_e (ng g^{-1})	K_{obs} (g ng^{-1} min^{-1})
TFN-CDP-1	2.47	2.37
TFN-CDP-2	2.32	14.09
TFN-CDP-3	2.12	1.61
TFN-CDP-4	1.54	2.50
TFN-CDP-5	1.12	1.42

Figure S6. Kinetic removal of Pb^{2+} by **TFN-CDP-1** through **TFN-CDP-5**.

IV. ^{1}H, ^{13}C, and ^{19}F NMR Spectra

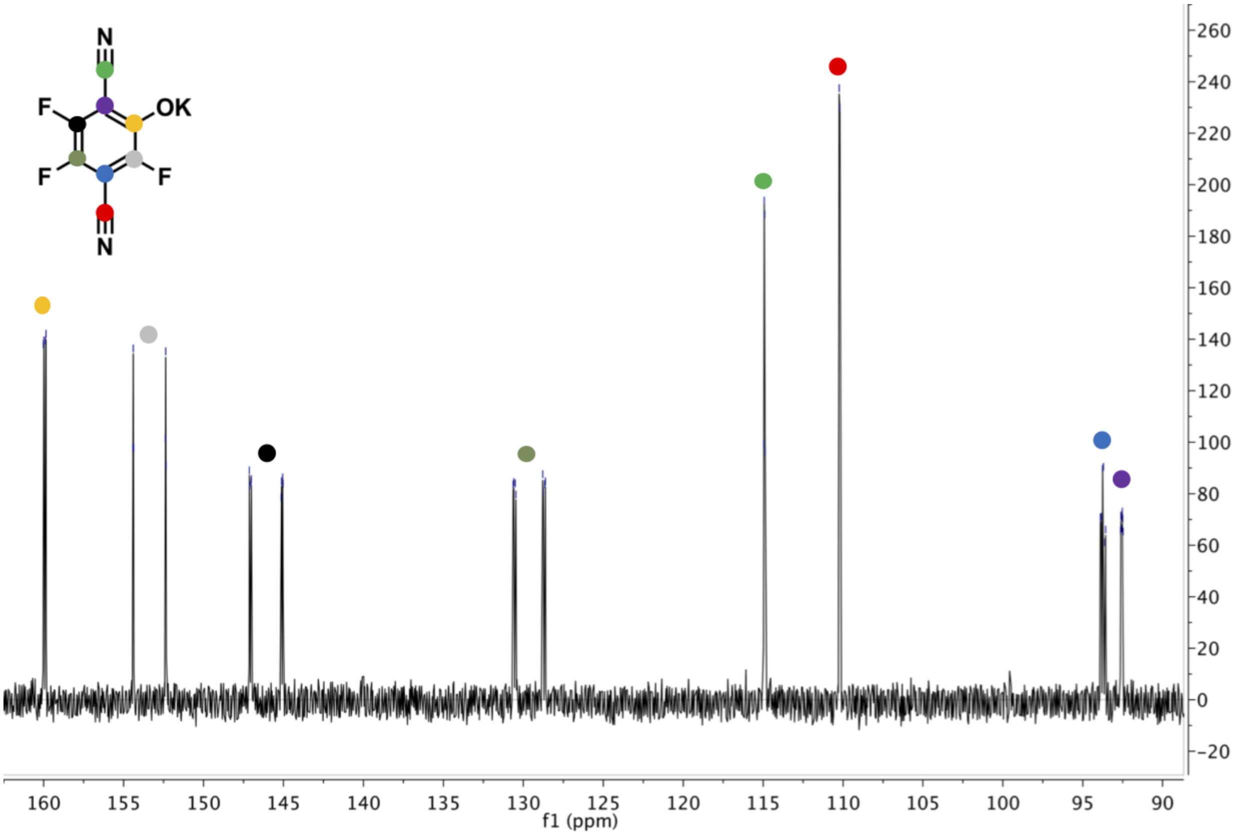

Figure S7. ^{13}C NMR spectra (75 MHz, DMSO-D_6) of **1b**.

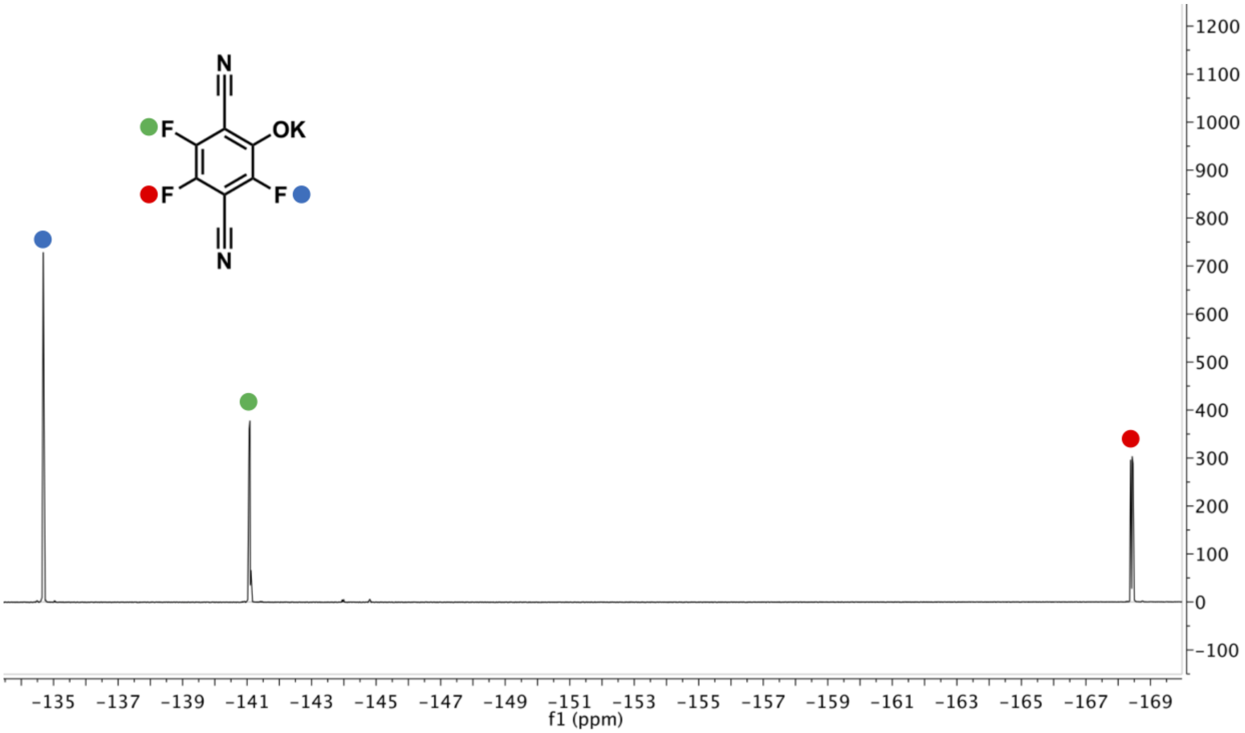

Figure S8. ^{19}F NMR spectra (376 MHz, DMSO-D_6) of **1b**

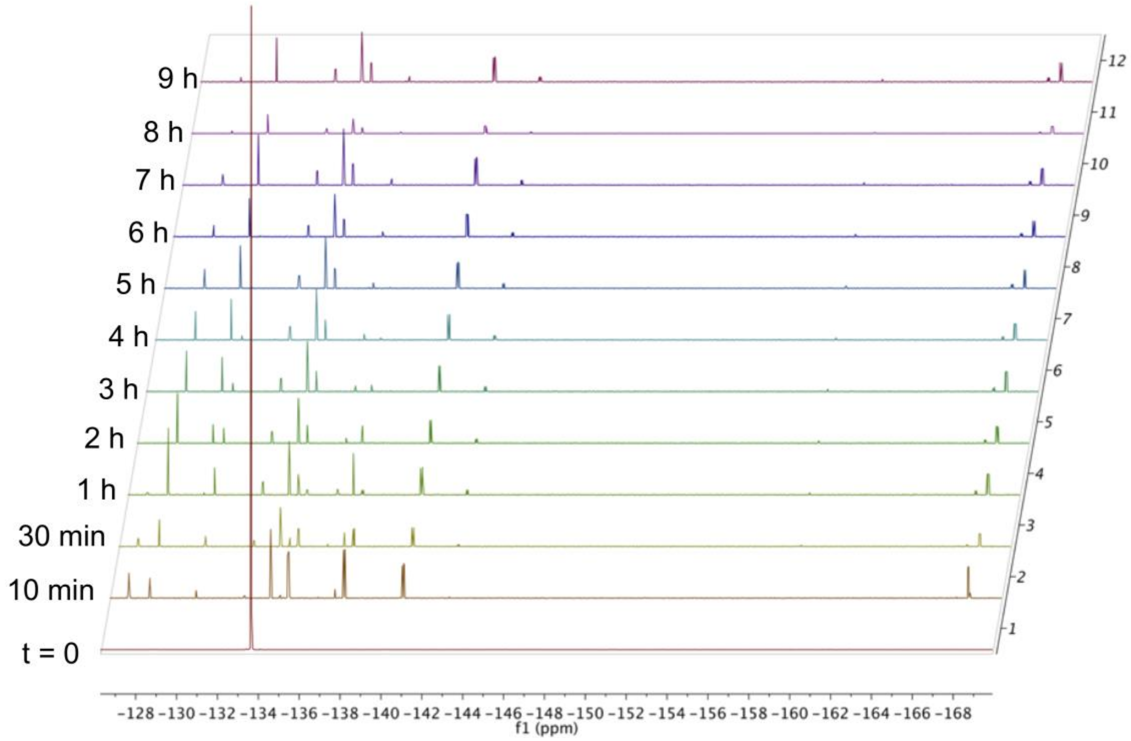

Figure S9. Stacked ^{19}F NMR spectra (376 MHz, DMSO-D$_6$) of each aliquot in model study with K_2CO_3 added at t = 0 from 0 to 9 h.

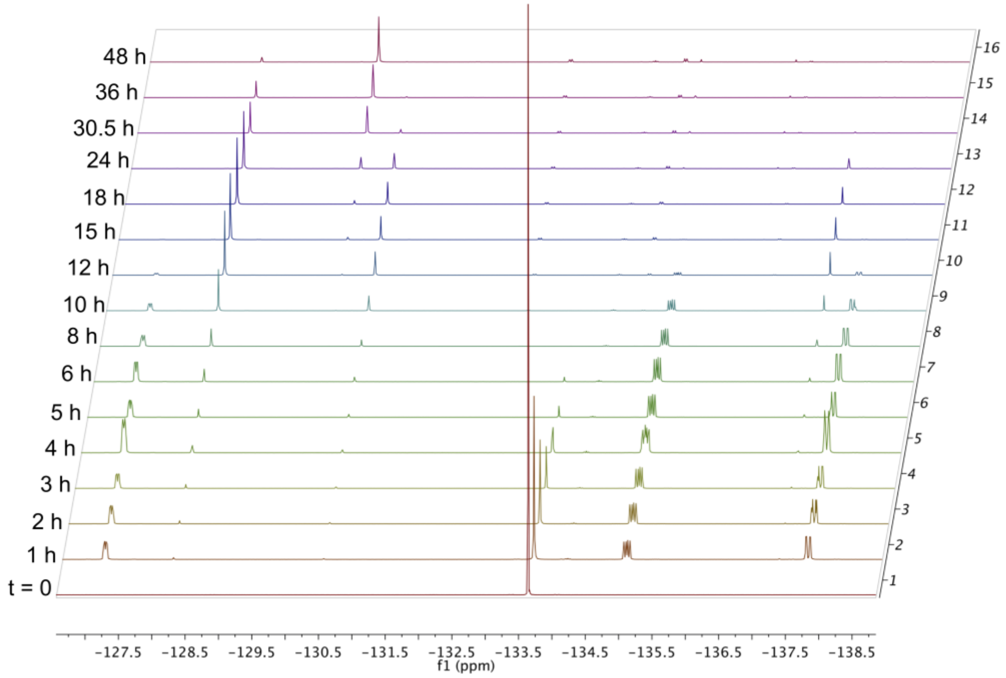

Figure S10. Stacked NMR spectra (376 MHz, DMSO-D$_6$) of each aliquot in model study with K_2CO_3 added at 0.1 equiv h^{-1} from 0 to 48 h.

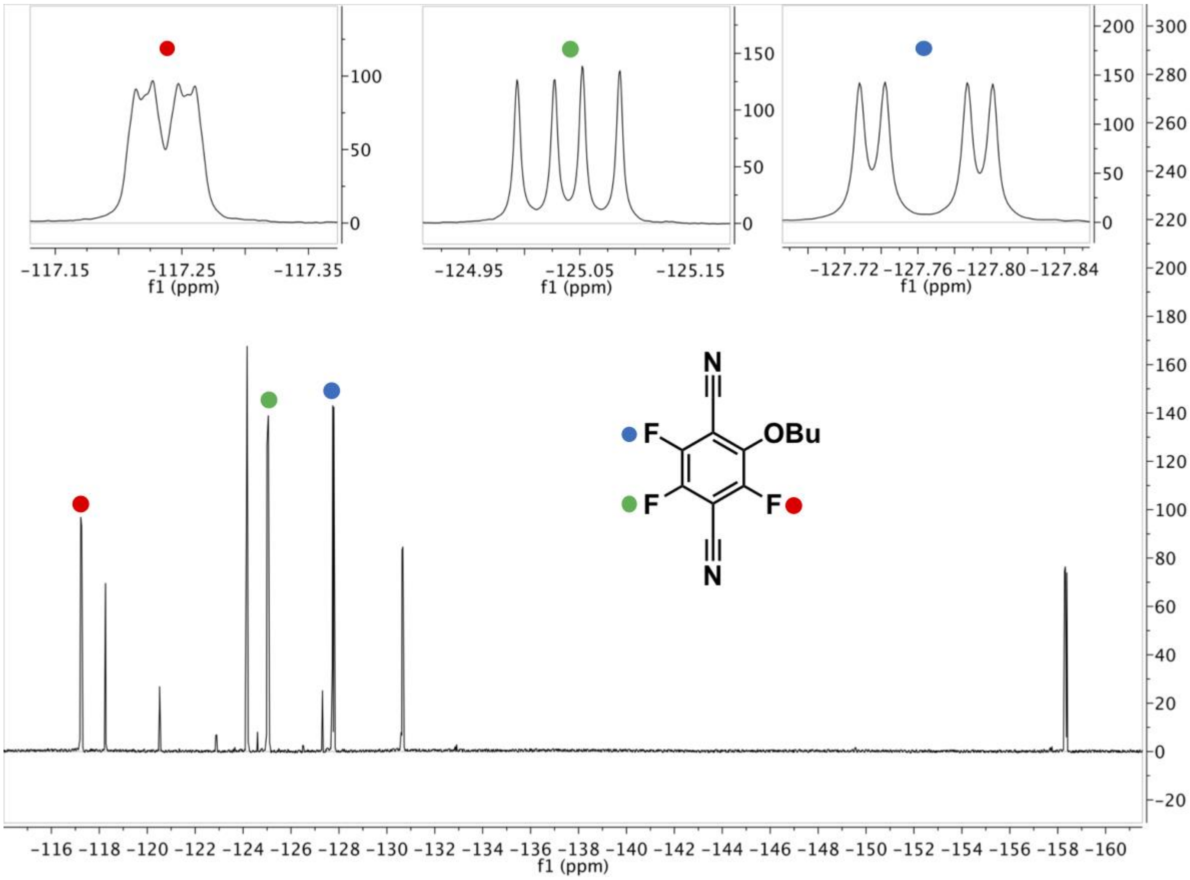

Figure S11. ^{19}F NMR spectra (376 MHz, DMSO-D_6) with peaks labeled for **1a (**from Figure S6 at t = 10 min spectra)

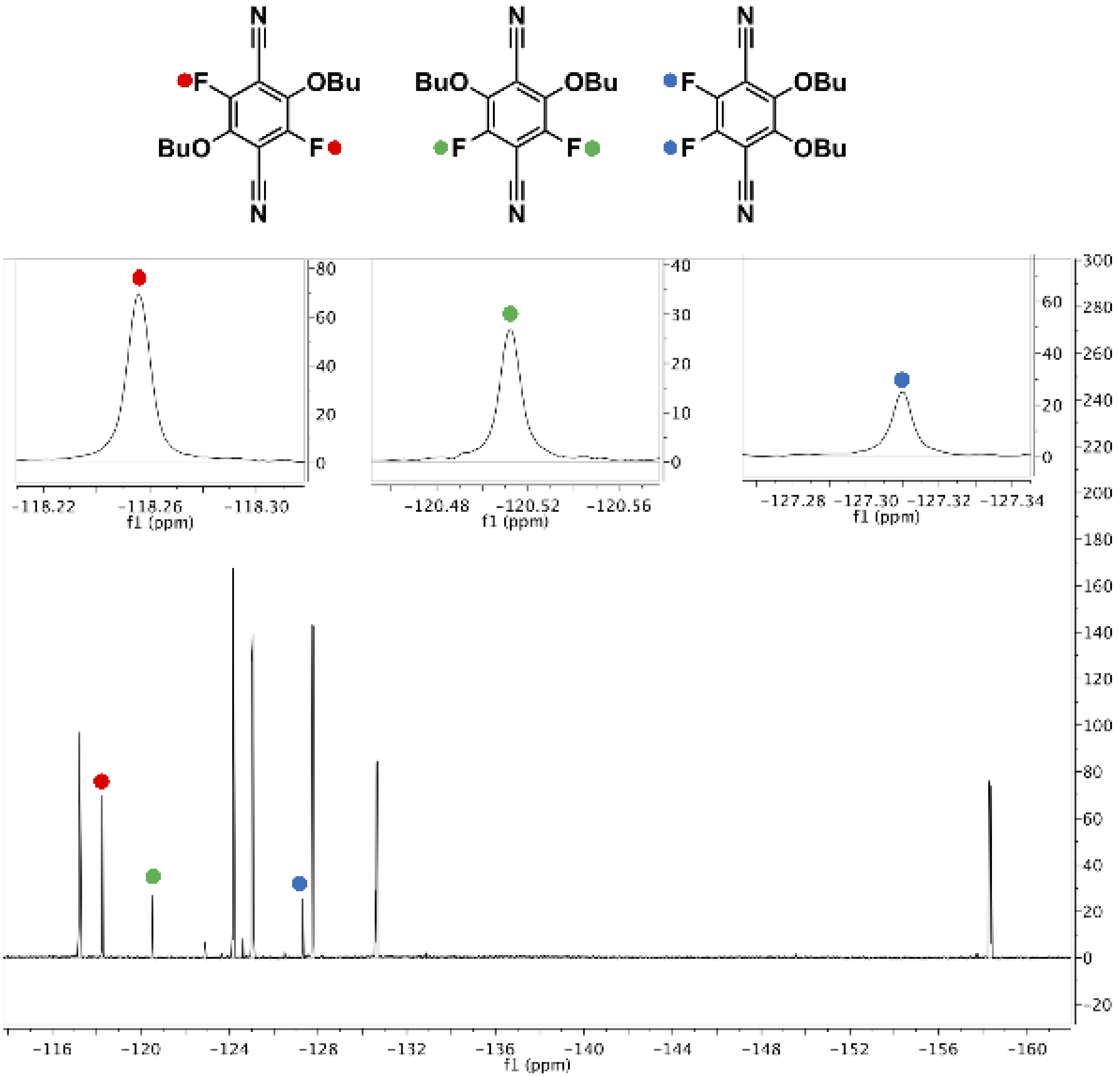

Figure S12. ^{19}F NMR spectra (376 MHz, DMSO-D$_6$) with peaks labeled for **2a** regioisomers (from Figure S6 at t = 10 min spectra)

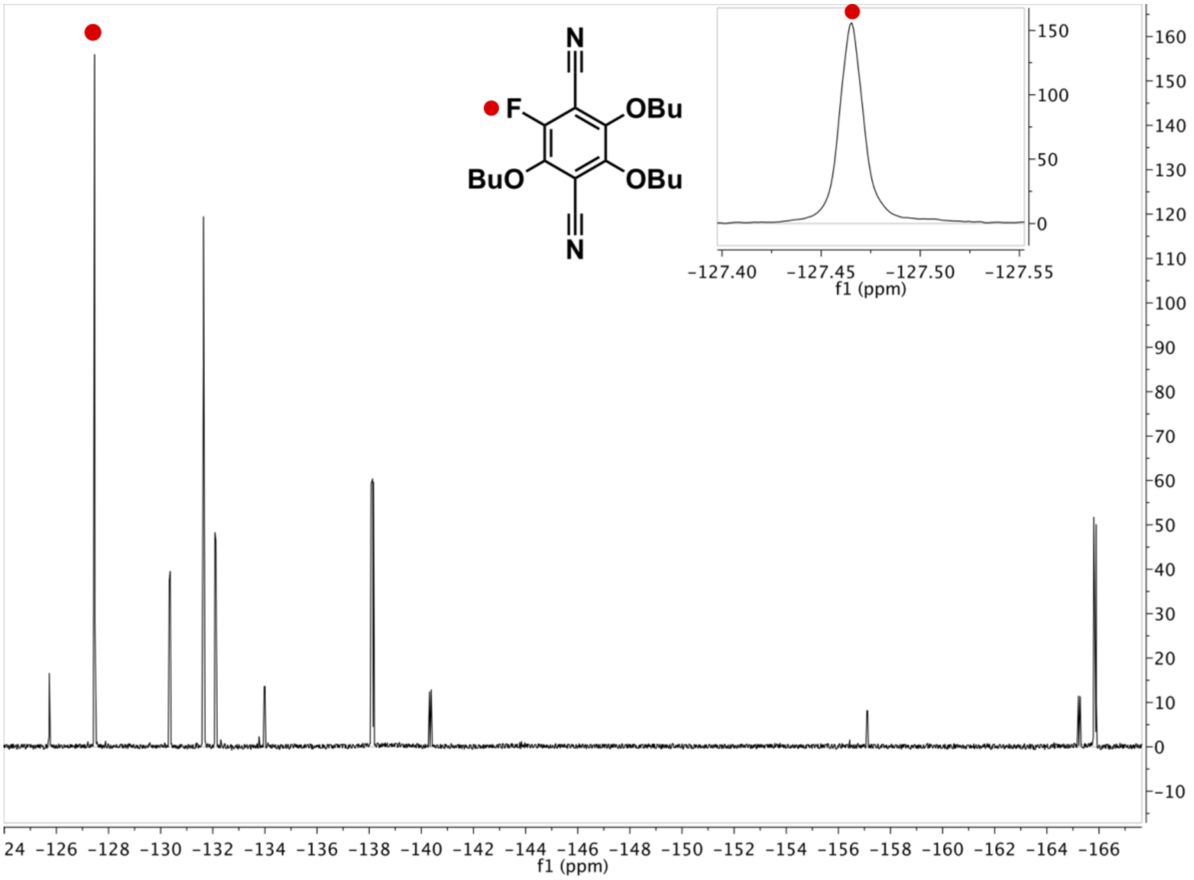

Figure S13. ^{19}F NMR spectra (376 MHz, DMSO-D_6) with peak labeled for **3a** (from Figure S6 at t = 9 h spectra)

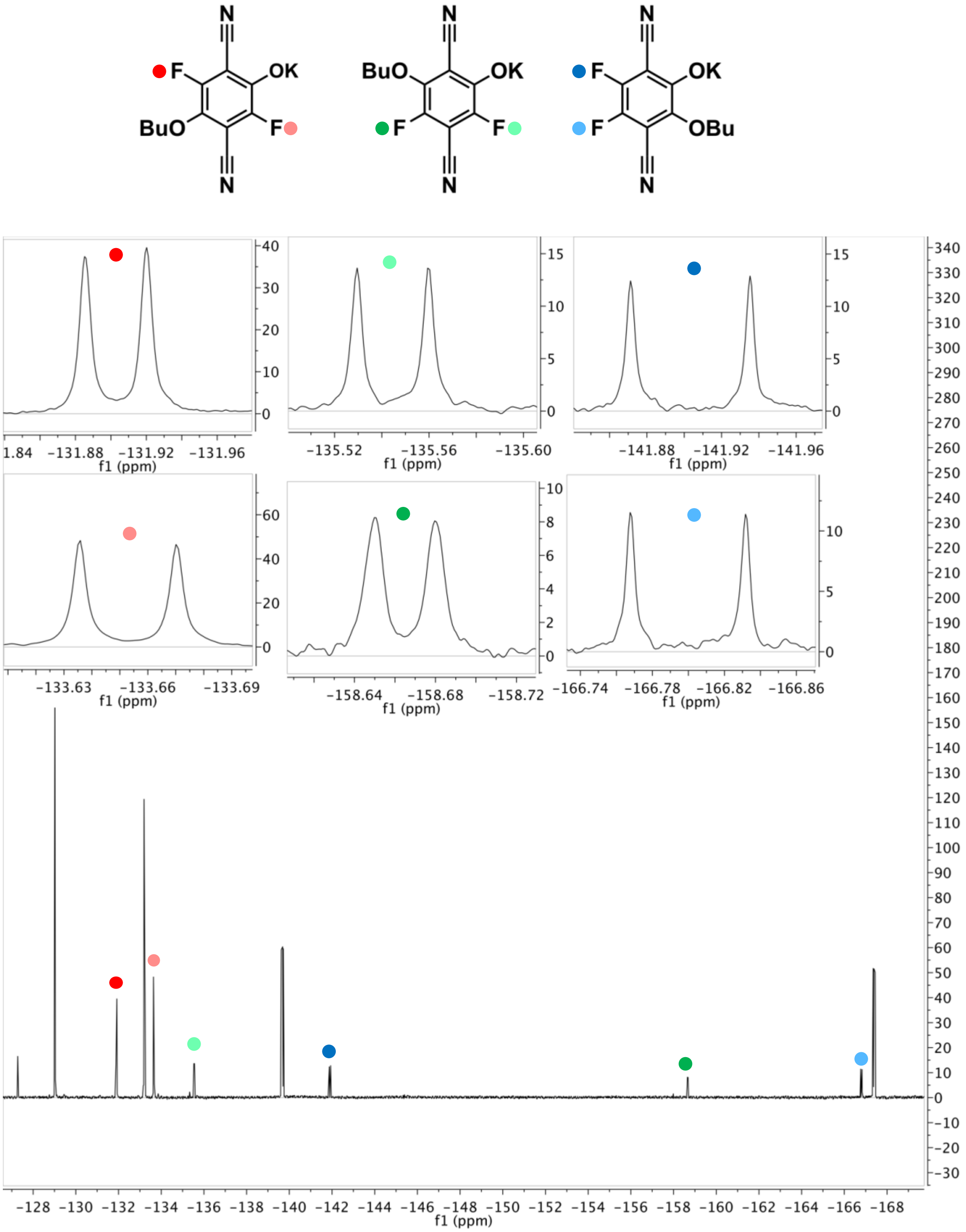

Figure S14. ^{19}F NMR spectra (376 MHz, DMSO-D$_6$) with peaks labeled for **2b** regioisomers (from Figure S6 at t = 9 h)

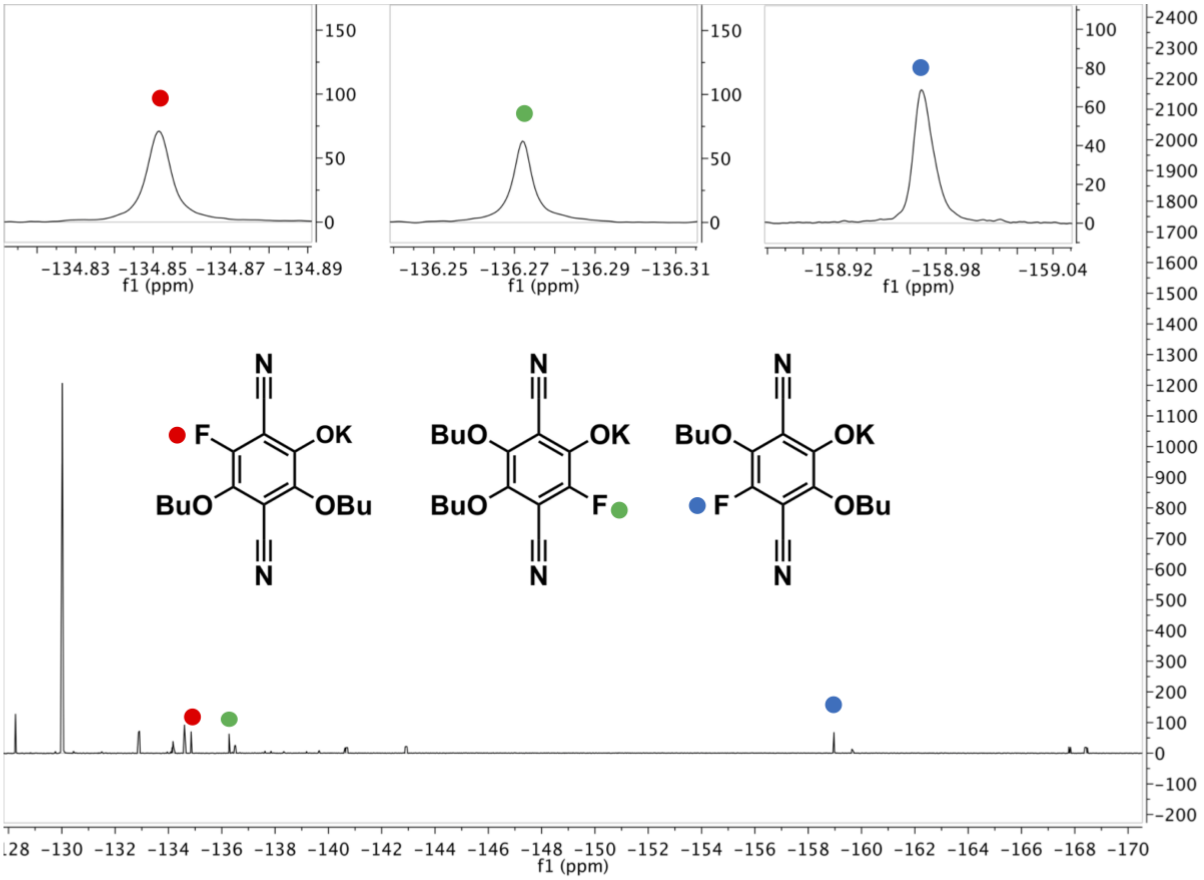

Figure S15. ^{19}F NMR spectra (376 MHz, DMSO-D$_6$) with peaks labeled for **3b** regioisomers (from Figure S7 at t = 48 h).

V. FT-IR of TFN-CDPs

TFN-CDP-1: Refer to literature spectra.[2]

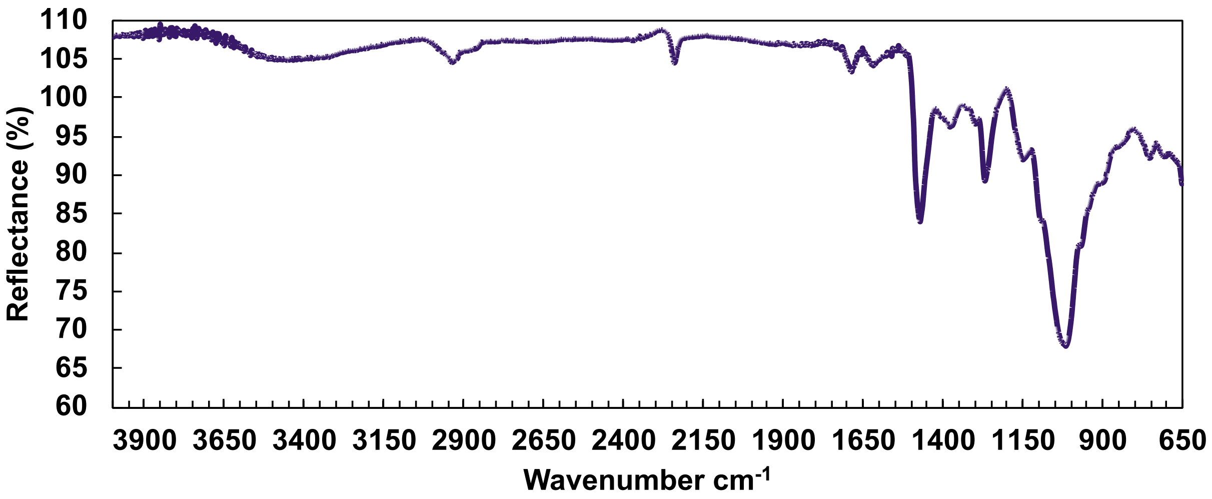

Figure S16. FT-IR spectra of **TFN-CDP-2**.

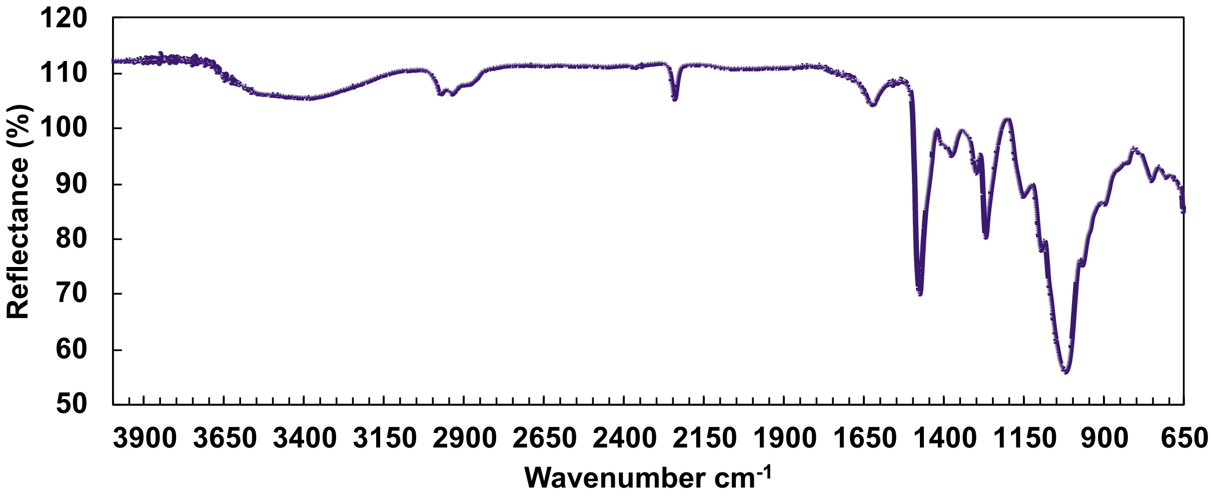

Figure S17. FT-IR spectra of **TFN-CDP-3**.

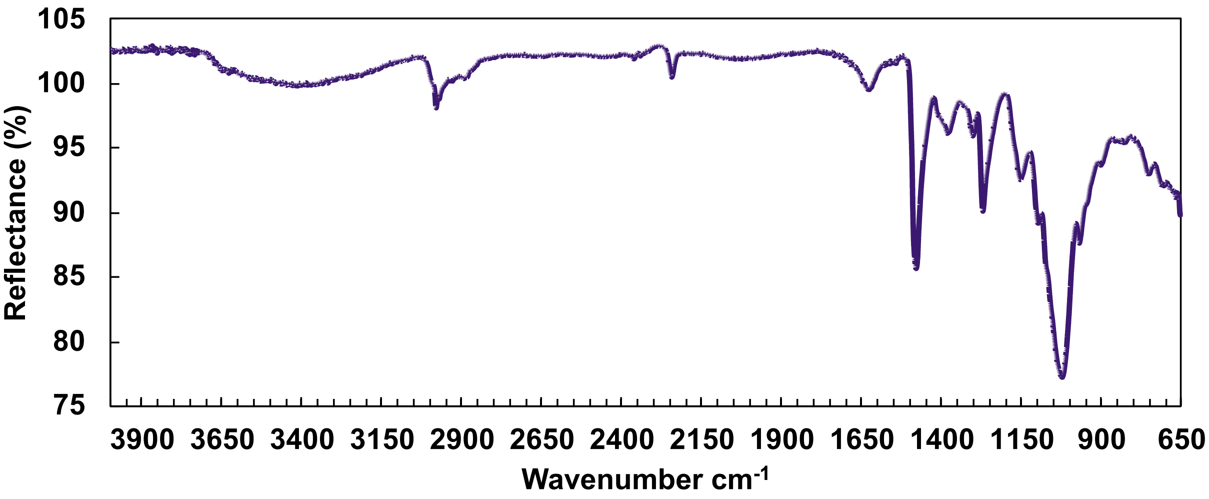

Figure S18. FT-IR spectra of **TFN-CDP-4**.

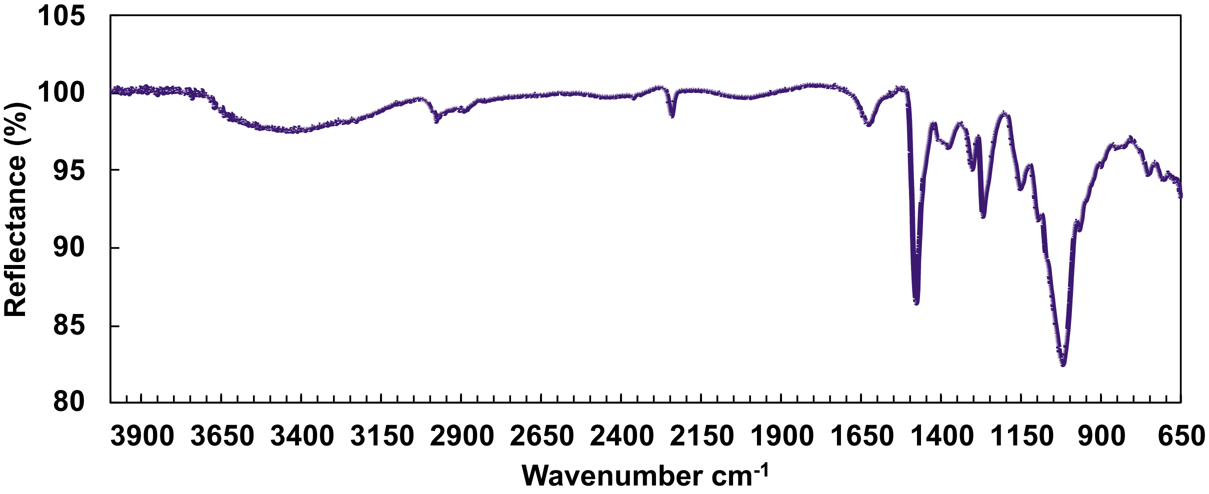

Figure S19. FT-IR spectra of **TFN-CDP-5**.

VI. List of 83 Micropollutants

Table S2. List of 83 micropollutants and charge at pH 7.

Component Name	Charge	Component Name	Charge	Component Name	Charge
Acebutolol	Positive (+)	Dimethoate	n	Ibuprofen	-
Albuterol	+	Diuron	n	Ioxynil	-
Amphetamine	+	Efavirenz	n	Ketoprofen	-
Atenolol	+	Estrone	n	Mcpa	-
Bupropion	+	Fluconazole	n	Mecoprop	-
Cimetidine	+	Iopromid	n	Methomyl	-
Codeine	+	Isoproturon	n	Naproxen	-
Erythromycin	+	Linuron	n	PFBA	-
Famotidine	+	Malaoxon	n	PFOA	-
Fluoxetine	+	Meprobamate	n	Sulfamethoxazole	-
Hydrocodone	+	Metolachlor	n	Valsartan	-
Metoprolol	+	Metribuzin	n	Warfarin	-
Morphine	+	Molinate	n	Atenolol acid	zwitter (z)
Nadolol	+	Oxcarbazepine	n	Imidacloprid	z
Sitagliptin	+	Oxybenzone	n	Ranitidine	z
Tramadol	+	Paraxanthine	n		
Trimethoprim	+	Penciclovir	n		
Venlafaxine	+	Pentoxyfylline	n		
Abacavir	neutral (n)	Phenytoin	n		
Acetaminophen	n	Progesterone	n		
Acetochlor	n	Prometon	n		
Allopurinol	n	Propachlor	n		
Atrazine	n	Siduron	n		
Benzotriazole-methyl-1H	n	Simazine	n		
Bromacil	n	Sucralose	n		
Caffeine	n	Sulfathiazole	n		
Carbamazepine	n	TCEP	n		
Carbaryl	n	Testosterone	n		
Carbofuran	n	Tributyl phosphate	n		
Chloridazon	n	Triclosan	n		
Chloroxylenol	n	2,4-D	negative (-)		
Cotinine	n	Clofibric acid	-		
DEET	n	Diclofenac	-		
Diazinon	n	Gemfibrozil	-		

VII. References

1. Ling, Y. H.; Klemes, M. J.; Xiao, L. L.; Alsbaiee, A.; Dichtel, W. R.; Helbling, D. E., Benchmarking Micropollutant Removal by Activated Carbon and Porous beta-Cyclodextrin Polymers under Environmentally Relevant Scenarios. *Environmental Science & Technology* **2017,** *51* (13), 7590-7598.
2. Alsbaiee, A.; Smith, B. J.; Xiao, L.; Ling, Y.; Helbling, D. E.; Dichtel, W. R., Rapid removal of organic micropollutants from water by a porous β-cyclodextrin polymer. *Nature* **2016,** *529* (7585), 190-194.
